# Distinguishing Old From New Referents During Discourse Comprehension: Evidence From ERPs and Oscillations

**DOI:** 10.3389/fnhum.2019.00398

**Published:** 2019-11-14

**Authors:** Mante S. Nieuwland, Cas W. Coopmans, Rowan P. Sommers

**Affiliations:** ^1^Max Planck Institute for Psycholinguistics, Nijmegen, Netherlands; ^2^Donders Institute for Brain, Cognition and Behaviour, Nijmegen, Netherlands; ^3^Centre for Language Studies, Radboud University, Nijmegen, Netherlands

**Keywords:** anaphora and coreference resolution, EEG and ERP, time-frequency analysis, N400 and P600, gamma and theta activity, beta activity, old/new effect, lexical repetition

## Abstract

In this EEG study, we used pre-registered and exploratory ERP and time-frequency analyses to investigate the resolution of anaphoric and non-anaphoric noun phrases during discourse comprehension. Participants listened to story contexts that described two antecedents, and subsequently read a target sentence with a critical noun phrase that lexically matched one antecedent (‘old’), matched two antecedents (‘ambiguous’), partially matched one antecedent in terms of semantic features (‘partial-match’), or introduced another referent (non-anaphoric, ‘new’). After each target sentence, participants judged whether the noun referred back to an antecedent (i.e., an ‘old/new’ judgment), which was easiest for ambiguous nouns and hardest for partially matching nouns. The noun-elicited N400 ERP component demonstrated initial sensitivity to repetition and semantic overlap, corresponding to repetition and semantic priming effects, respectively. New and partially matching nouns both elicited a subsequent frontal positivity, which suggested that partially matching anaphors may have been processed as new nouns temporarily. ERPs in an even later time window and ERPs time-locked to sentence-final words suggested that new and partially matching nouns had different effects on comprehension, with partially matching nouns incurring additional processing costs up to the end of the sentence. In contrast to the ERP results, the time-frequency results primarily demonstrated sensitivity to noun repetition, and did not differentiate partially matching anaphors from new nouns. In sum, our results show the ERP and time-frequency effects of referent repetition during discourse comprehension, and demonstrate the potentially demanding nature of establishing the anaphoric meaning of a novel noun.

## Introduction

All nouns have a general meaning, maybe even multiple general meanings, but they acquire a particular, referential meaning when used to refer to someone or something in the world. This flexible use of language and memory yields incredible expressive power for communicating information about the world (e.g., Clark and Murphy, 1982; Martinich, 1985; Gibson and Pearlmutter, 2011), but also harbors a potential mapping problem for language comprehenders: different words like ‘martian’ and ‘alien’ can have the same referent, and the same word can have different potential referents, such as ‘the alien’ when there are multiple aliens in the context. To examine how people solve such mapping problems, we compared electrophysiological brain responses [event-related potentials (ERPs) and oscillatory activity] to referring expressions that have either one, two or no suitable referent in the linguistic context and that may differ in form (and general meaning) from their referent.

Our study investigates the comprehension of expressions that refer to a previously mentioned referent in the discourse context, i.e., *anaphoric reference* to a linguistic antecedent (e.g., Garnham, 2001; Almor and Nair, 2007). Psycholinguistic theories stipulate the importance of general memory representations and processes during anaphor resolution (e.g., Garrod and Sanford, 1977; Gernsbacher, 1989; McKoon and Ratcliff, 1998; Myers and O’Brien, 1998). Such theories often distinguish an initial activation phase, wherein anaphors are thought to reactivate antecedents from a memory representation of the context (including the described referents), and a subsequent integration phase wherein the reactivated representation is integrated with the unfolding representation of the narrated event. Our main interest in this paper is antecedent activation, which is viewed as a memory-based process in which semantic and syntactic content of an anaphor serves as a memory cue to the antecedent. This process entails the recognition of the anaphor as an instantiation of the antecedent – even when they differ in linguistic form – through the computation of a similarity/identity relation between the two words. This computation gives the language system both great flexibility and speed, by enabling efficient reactivation of semantically complex concepts (e.g., ‘Boris Johnson’), either by other complex concepts (‘blonde haired Brexiteer’) or by minimal-content pronouns (‘he’). The ease with which people understand noun phrase anaphors depends on content overlap of the anaphor with the intended referent relative to other antecedents (e.g., Garrod and Sanford, 1977, 1982; Krahmer and Deemter, 1998; Almor, 1999; Van Gompel et al., 2004; Pyke, 2007). Repeated noun phrase anaphors are easier to resolve than anaphors that only partially match an antecedent (e.g., McKoon and Ratcliff, 1980; Tyler, 1983; Walker and Yekovich, 1987), e.g., ‘the alien’ referring to an alien/a martian^1^. An anaphor whose semantic content does not distinguish between antecedents, e.g., ‘the alien’ in a story about two aliens, is referentially ambiguous. A preceding determiner may already hint at whether the upcoming noun is anaphoric (e.g., Garrod and Sanford, 1977; Clark and Sengul, 1979; Garnham, 1989), with the definite determiners ‘the’ heralding an anaphoric noun phrase and the indefinite determiner ‘a’ heralding a novel, non-anaphoric noun phrase. However, definite noun phrases sometimes introduce a new referent (e.g., Heim, 1982; Fraurud, 1990; Garrod et al., 1994; Poesio and Vieira, 1998; Gundel et al., 2001; Pyke, 2007; Pyke et al., 2007a, b), and people can use the semantic content of a definite noun as a basis to introduce a novel referent when required, e.g., ‘the alien’ when the context only mentioned astronauts. This process is sometimes referred to as discourse updating (e.g., Burkhardt, 2006), which is related to, yet distinct from the integration process by which people process discourse-level meaning (e.g., Coopmans and Nieuwland, 2019). In other words, processes involved in noun phrase anaphor resolution must distinguish old from new referents, and may do so partly relying on *memory* processes (for a review and computational account, see Pyke, 2007). To address this issue, the current study investigates whether old and new noun phrase referents elicit distinct neural responses, as measured with ERPs and time-frequency analysis.

### Noun Phrase Anaphors and ERPs

Noun phrase anaphors have been associated with several distinct ERP effects, in particular with modulations of the N400, the Late Positive Component (LPC), and the Nref effect. The N400 component is a negative ERP deflection that peaks approximately 400 ms after word onset and is maximal at centroparietal electrodes (Kutas and Hillyard, 1980). The N400 reflects semantic processing and its amplitude is modulated by the relationship between the meaning of a word and its context (Kutas and Hillyard, 1980, 1984; for review, see Kutas and Federmeier, 2011). Words whose meaning is easier to access based on the context typically elicit reduced N400 amplitude compared to words whose meaning is unrelated to the context (Kutas and Federmeier, 2011). Compatible with such findings, noun phrase anaphors that are either repeated from the context or that are contextually implied (‘the conductor’ in a context describing an orchestra) elicit reduced N400 amplitude compared to novel, unrelated noun phrases (e.g., Burkhardt, 2006, 2007). Such N400 modulations may reflect the ease with which the meaning of the anaphor is activated as a function of the context (e.g., Kutas and Federmeier, 2011), and need not reflect higher-level processes such as discourse updating or integration. While recent studies suggest that N400 activity can arise from a cascade of processes that activate and integrate word meaning with context into a sentence-level meaning (e.g., Baggio and Hagoort, 2011; Baggio, 2019; Nieuwland et al., 2019), some studies have failed to observe updating- or integration-related effects on the N400 and found them on a later positive-going ERP component, the LPC (e.g., Burkhardt, 2006, 2007; Delogu et al., 2019). For example, Burkhardt (2006) reported that contextually implied and novel definite referents (‘the conductor’ when the context does or does not describe an orchestra, respectively) elicit a similar post-N400, LPC when compared to a repeated noun phrase anaphor. Burkhardt concluded that the LPC effect reflected the costs of updating a discourse representation with an additional referent (for such costs observed in behavioral studies, see, for example, Murphy, 1984; cf., Pyke, 2007). Subsequent studies found compatible results with related manipulations (Burkhardt, 2007; Schumacher and Hung, 2012). However, the nature and generalizability of this reference-related LPC effect remains to be established. One study with a similar manipulation did not report any LPC modulation (Yang et al., 2007). And while one recent study with repeated proper name anaphors also reported enhanced LPC for new names (Coopmans and Nieuwland, 2019), two other studies with proper names reported a reverse LPC pattern (Van Petten et al., 1991; Swaab et al., 2004). For example, in a study on natural text comprehension, Van Petten et al. (1991) reported enhanced LPC amplitude for repeated proper names compared to novel names, and suggested that these effects reflect the retrieval of semantic information associated with known names^2^.

Whereas the semantic relationship between an anaphor and its context can modulate the N400 (and LPC), the referential relationship between an anaphor and its context can elicit an LPC effect or yet another ERP effect. Referentially ambiguous anaphors, like ‘the alien’ when two different aliens were mentioned in the context, or the pronoun ‘he’ without a male antecedent in the sentence, elicit a sustained, frontal negativity compared to non-ambiguous anaphors (the Nref effect; for reviews, see Van Berkum et al., 2007; Nieuwland and Van Berkum, 2008b). The Nref effect can start at about 200–300 ms after word onset (not unlike an N400 effect, at least for written language comprehension), and has been obtained with noun phrases (e.g., Van Berkum et al., 1999a, 2003; Nieuwland et al., 2007; Nieuwland and Van Berkum, 2008a), pronouns (e.g., Nieuwland and Van Berkum, 2006; Nieuwland, 2014; Karimi et al., 2018), noun phrase ellipsis (e.g., Martin et al., 2012), and proper names (e.g., Coopmans and Nieuwland, 2019). While the onset latency of the Nref suggests that it indexes processes that rapidly link expressions to potential referents, the sustained nature of this effect suggests that inability to resolve reference may have a prolonged impact on comprehension (see Nieuwland et al., 2007; Nieuwland and Martin, 2017).

### Anaphora and Neural Oscillations

ERPs are the most common dependent measure in electrophysiological research on language comprehension, but some studies have instead or additionally examined neural oscillatory responses, measured with time-frequency analysis. Oscillatory activity reflects the synchronization and desynchronization of neural populations, i.e., the transient coupling or uncoupling of functional cell assemblies (e.g., Engel et al., 2001; Buzsáki and Draguhn, 2004). ERPs and oscillatory responses are complementary electrophysiological measures, because whereas ERP analysis can only detect activity that is both time- and phase-locked to stimulus onset, time-frequency analysis can detect activity that is time-locked only^3^. To date, only a handful of studies have applied time-frequency analysis to examine reference processing (Van Berkum et al., 2004; Heine et al., 2006; Boudewyn et al., 2015^4^; Meyer et al., 2015; Nieuwland and Martin, 2017; Coopmans and Nieuwland, 2019).

Heine et al. (2006) reported that pronouns with low-frequency antecedent nouns elicit reduced power in the theta (4–7 Hz) range compared to pronouns with high-frequency antecedents. They argued that pronoun resolution is relatively easy for low-frequency words because they capture elevated attention. Consistent with a role for memory processes in pronoun resolution, source analyses (albeit based on low resolution, 27-channel EEG data) suggested a contribution from the parahippocampal gyrus to the observed theta effect.

Meyer et al. (2015) reported that pronouns with antecedents that were embedded in a subordinate clause elicit enhanced theta power compared to pronouns referring to non-embedded antecedents, and source analysis suggested contributions from left-frontal, left-parietal, and bilateral-inferior-temporal cortices (based on 64-channel data). Meyer and colleagues argued that embedded antecedents were harder to retrieve from verbal working memory compared to non-embedded antecedents.

In other words, both Heine et al. (2006) and Meyer et al. (2015) took enhanced theta power to index difficulty with reactivating or retrieving an antecedent from memory, in line with the literature on theta effects and verbal and non-verbal working memory retrieval (e.g., Bastiaansen and Hagoort, 2003; Jacobs et al., 2006). However, it is unclear whether the reported theta effects were truly oscillatory in nature and distinct from phase-locked activity that also yields an associated ERP effect.

Two other studies report effects of reference processing in the gamma (>30 Hz) frequency range but not in the theta range. An unpublished study by Van Berkum et al. (2004) reported increased gamma power (40–55 Hz) range for pronouns with a single matching antecedent (e.g., ‘she’ in a sentence with one male and one female antecedent) compared to pronouns with two or zero matching antecedents (‘she’ in a sentence with either two female or two male antecedents, respectively). A study by Nieuwland and Martin (2017) re-analyzed four EEG datasets that had initially been collected for ERP analysis (Nieuwland and Van Berkum, 2006; Nieuwland et al., 2007; Martin et al., 2012; Nieuwland, 2014). In each dataset they observed increased gamma power for referentially successful expressions (pronouns, noun phrases, ellipsis that matched a single antecedent) compared to referentially problematic expressions (with either two matching antecedents or no matching antecedent). In one of those four studies, they compared the oscillatory response to a matching pronoun with that to a mismatching, ambiguous pronoun (e.g., “The boy said that he/she would win the race”). They found a brief gamma power increase in the 35–45 Hz range between 400 and 600 ms after pronoun onset. Beamformer source analysis (64-channel data) suggest contributions from left posterior parietal cortex, a brain region that is thought to be involved in recognition memory (Cabeza et al., 2008). They also observed a more extended gamma power increase in the 60–80 Hz range between 500 and 1000 ms after pronoun onset, with source analysis suggesting a contribution from left inferior frontal gyrus, and brain region that is thought to be involved in sentence-level unification/integration processes (e.g., Hagoort, 2005; Hagoort and Indefrey, 2014). Based on these findings, Nieuwland and Martin (2017) argued that the observed gamma-band power increases reflect successful referential binding and resolution, which links incoming information to antecedents through an interaction between the brain’s recognition memory networks and fronto-temporal language network.

In a recent study on comprehension of proper name anaphors, Coopmans and Nieuwland (2019) observed effects in both the theta and gamma frequency range. Their participants read story contexts that described characteristics of two people (e.g., “John and Peter are the best players in the football team”), followed by a target sentence containing a repeated or novel proper name that was either congruent or incongruent with the discourse context (e.g., “The top scorer of the team was John with thirty goals in total”). Repeated names elicited increased theta power compared to new names, which may have originated from anterior temporal regions (based on beamformer source analysis of 64-channel data), and a weak effect in the 40–55 Hz gamma range (see also Van Berkum et al., 2004). Discourse-congruent names elicited increased gamma power (60–80 Hz) compared to incongruent names in the 500–1000 ms time window, with source analysis suggesting a contribution from left frontal cortex.

In sum, reference processing thus far has been associated with modulations of theta and gamma activity. However, the available studies report mixed results, which may have to do with differences in type of linguistic expression (pronoun, noun phrase, proper name) and experimental manipulation (difficulty with retrieving an antecedent, referential ambiguity, comparing old, anaphoric names with new names). Heine et al. (2006) and Meyer et al. (2015) investigated pronouns that had uniquely identifiable antecedents but differed in the extent to which the antecedent was easily retrieved from memory, whereas Nieuwland and Martin (2017) compared ambiguous to unambiguous anaphors, and Coopmans and Nieuwland (2019) compared anaphoric to non-anaphoric proper names that were coherent or incoherent with the preceding discourse. The type of linguistic expression may matter in particular for modulations of theta activity, because theta activity can be modulated by a word’s semantic meaning (e.g., Bastiaansen et al., 2005, 2008).

### The Present Study

The present EEG study investigated how people establish anaphoric meaning for noun phrases, which contain more semantic content than pronouns and proper names and therefore allow an investigation of how people can use semantic memory representations (i.e., word meaning) to resolve anaphoric reference (e.g., Garrod and Sanford, 1977; Garnham, 1989). This semantic richness raises the question of whether or to what extent anaphoric noun phrases are resolved through similar processes as other types of anaphors. Our participants listened to two-sentence story contexts followed by a written sentence that contained a target noun. These stories appeared in one of four conditions that only differed in the two antecedents described in the first sentence (see Table 1). Due to these differences, the target noun was either a given or ‘old’ anaphor (lexically identical to one of the two antecedents), an ‘ambiguous’ anaphor (lexically identical to both antecedents), a ‘partial-match’ anaphor (lexically different from both antecedents but close enough in meaning to one of the antecedents to allow an anaphoric interpretation, as indicated in a norming pre-test), or a ‘new’ noun (lexically and semantically different enough from both antecedents such that a novel referent must be introduced). After each story, the participants used a button press to indicate whether the target sentence contained an anaphoric noun phrase or not (old/new judgment). While this task requires meta-linguistic judgments and is therefore not representative for naturalistic comprehension, we included it in order to separate trials in which participants arrived at the intended interpretation from trials where they did not (as is also done in studies on recognition memory).

**TABLE 1:** Example stimulus item in Dutch, containing all four conditions.

**Condition**	**First spoken context sentence**	**Second spoken context sentence**	**Written target sentence**
Old	Een oude receptioniste en een jonge sollicitant plannen een nieuwe afspraak. *An old receptionist and a young applicant are planning a new appointment.*	De afspraak vindt in mei plaats. *The appointment will take place in May.*	Na het plannen schrijft de **receptioniste** direct de datum op. *After planning, the receptionist immediately writes down the date.*
Ambiguous	Een oude receptioniste en een jonge receptioniste plannen een nieuwe afspraak. *An old receptionist and a young receptionist are planning a new appointment.*		
Partial	Een oude baliemedewerker en een jonge sollicitant plannen een nieuwe afspraak. *An old desk clerk and a young applicant are planning a new appointment.*		
New	Een oude sollicitant en een jonge sollicitant plannen een nieuwe afspraak. *An old applicant and a young applicant are planning a new appointment.*		

*Approximate English translation is provided below each sentence. The critical word is printed in bold for presentation purposes only. All stimuli available via our OSF page https://osf.io/uak8g.*

For this experimental design, we derived hypotheses from memory-based theories of anaphor resolution (e.g., Myers and O’Brien, 1998), which distinguish an early phase of memory activation from subsequent discourse updating and integration. We hypothesized that activity in the early phase primarily depends on the ease with which word meaning can be activated, which is easiest for repeated nouns. For the ERP analysis, we expected to observe this phase in N400 activity (e.g., Kutas and Federmeier, 2011), with smaller (less negative) N400 ERPs for old and ambiguous anaphors compared to new nouns and partial-match anaphors (i.e., a lexical repetition effect on the N400, e.g., Van Petten et al., 1991; Besson et al., 1992; Swaab et al., 2004). We also expected smaller N400s for partial-match anaphors compared to novel nouns, because the semantic meaning of partial-match anaphors is more strongly related to the context and therefore more easily activated than that of novel nouns (Kutas and Federmeier, 2000). In our time-frequency analysis, we tested for complementary effects in the theta- and gamma-band, which are strongly associated with memory processes. We expected to observe enhanced theta (and low gamma) power for anaphoric nouns compared to new nouns (see Nieuwland and Martin, 2017, for discussion). Such a pattern would be compatible with the proper name effects recently observed by Coopmans and Nieuwland (2019), and consistent with theta and gamma band effects associated with successful recognition in memory research. However, this hypothesis disregards the association between theta activity and activation of semantic representations (e.g., Bastiaansen et al., 2005, 2008; Piai et al., 2016), which is why we also considered an alternative possibility: if theta power tracks the amount of semantic activation (e.g., Bastiaansen et al., 2005), new nouns could elicit enhanced theta power compared to old nouns.

Activity in the later, post-N400 time-window may be associated with either repetition or with discourse-level processes^5^. For example, we considered the possibility that anaphoric nouns would elicit larger LPCs than novel nouns (Van Petten et al., 1991; Swaab et al., 2004), although such a pattern for repeated referents has not yet been found for noun phrases. We also considered an alternative possibility, namely that new nouns would elicit larger LPCs than anaphors (which would suggest that this component indexes updating of the discourse representation to include a new referent; Burkhardt, 2006; Coopmans and Nieuwland, 2019). Furthermore, we expected ambiguous anaphors to elicit an Nref effect compared to non-ambiguous anaphors (Van Berkum et al., 1999a; Nieuwland et al., 2007; Nieuwland and Van Berkum, 2008a, b). For the time-frequency analysis, we expected enhanced high gamma (60–80 Hz) activity for anaphors compared to new nouns, possibly related to updating or integration processes (e.g., Nieuwland and Martin, 2017).

Of specific interest were the processes involved in resolving partially matching anaphors, which differ in form and meaning from the antecedent (e.g., baliemedewerker-receptioniste, desk clerk-receptionist, in Table 1). Previous literature suggests that such anaphors may be relatively difficult to resolve because they unexpectedly introduce new information (Garrod and Sanford, 1977; Garnham et al., 1997), which is atypical for anaphors. This violation of pragmatic principles may cause people to consider the possibility that a new referent is being introduced, and the resulting situation can only be resolved through an elaborative, anaphoric inference based on the semantic similarity of anaphor and antecedent. In such an account, old, new, and partially matching anaphors may elicit a difference in measures that index semantic activation (N400, possibly theta), but later measures could indicate whether the partially matching noun is temporarily processed as a new noun, by comparing the associated neural responses to responses elicited by new or old nouns, respectively. Alternatively, ambiguity regarding the anaphoric nature of partially matching nouns could lead to the type of Nref effect we expected for ambiguous nouns (Nieuwland, 2014).

## Materials and Methods

We pre-registered the number of participants and crucial elements of data processing and analysis on AsPredicted.org, available through the OSF pre-registration portal^6^. Procedures and analyses that were not pre-registered are designated as exploratory.

### Participants

We invited 41 participants (right-handed native-Dutch speakers who were free from known learning or language disorders) from the MPI participant pool (34 females, average age = 23.3 years, range = 19–32 years). All participants gave informed written consent to take part in the experiment, which was approved by the Ethics Committee for Behavioural Research of the Social Sciences Faculty at Radboud University Nijmegen in compliance with the Declaration of Helsinki. They received 18 euros for their participation. One participant did not finish the experiment and was replaced. For the ERP analysis, we excluded three participants due to low trial numbers (on average across conditions < 35 artifact-free trials with correct responses). For the time-frequency analysis, we excluded five participants due to low trial numbers.

### Stimuli

The entire set of stimuli consisted of 200 experimental and 50 filler mini stories in Dutch. Each mini story consisted of three sentences, of which the first sentence introduced two antecedents (persons or objects), and the third sentence contained a critical noun phrase that also denoted a person or object (see Table 1). The antecedents appeared in an indefinite conjoined noun phrase that included two prenominal adjectives and that either repeated the same noun (ambiguous and new condition) or contained different nouns (old and partial-match condition). The critical word (CW) in the third sentence was always a definite noun phrase without a prenominal adjective, was never the first or second word of the sentence, and was followed by exactly four additional words in the sentence.

Both the second context sentence and the target sentence were identical across conditions. The four conditions differed only in the two antecedents described in the first sentence, which determined the available co-referential relationships between the critical word and the antecedents. The critical word in the old condition was a repeated name anaphor, which was identical to and co-referential with one antecedent (*receptionist*-*receptionist)*. The ambiguous anaphor was identical to both antecedents. The partially matching anaphor was semantically overlapping or synonymous with only one of the antecedents (*desk clerk-receptionist*, we report semantic similarity values below), which were chosen such that the critical word would be a reasonably plausible anaphor for one antecedent. In the new condition, the critical word did not appear elsewhere in the context, and it had little semantic overlap with either antecedent to the extent that it would not be a plausible anaphor. We tried to write stories wherein the partially matching anaphor was related in meaning to the story context and to the antecedent and plausibly co-referential with the first antecedent, and wherein the novel noun was at least somewhat related in meaning to the story context but not plausibly co-referential and would therefore be interpreted as introducing a new referent. In both the given and the partial-match condition, the anaphor always referred to the first antecedent in the context sentence.

In an effort to optimize our stimulus set for these constraints, we performed a behavioral norming study on an initial set of 240 items. Twenty-four participants, who did not take part in the EEG experiment, each read 240 stories in the New, Old or Partial-Match condition, with conditions counterbalanced over three stimulus lists such that each participant saw the same number of items per condition and each item was seen in each condition equally often across participants. The participants read each story presented as a whole on the screen with the target word in boldface, and judged whether each target word referred back to someone or something in the story (‘old’) or whether it referred to someone or something new (‘new’). Based on the results, we selected the best 200 items, that is, items receiving responses most in line with our design (partial-matching and old anaphors considered ‘old’ and novel nouns considered ‘new’). Because we made further changes to the selected materials after the norming study, and because we also collected old/new judgments during the main EEG experiment (which are the most relevant behavioral data), the results of the stimulus norming test are not discussed here, but they can be found on our OSF page^7^.

For the final set of items, we confirmed that partially matching nouns were more semantically similar to the corresponding first antecedent than new nouns. We used semantic similarity scores obtained from ‘snaut’ (Mandera et al., 2017)^8^, using a word2vec-compatible ‘continuous bag of words’ (CBOW) model for Dutch lemmas, trained on the SONAR-500 corpus and an additional subtitle corpus. With the caveat that not all our words found a match in the corpus (155 partially matching nouns and 149 new nouns), partially matching nouns and their antecedents had a smaller semantic distance (i.e., were more semantically similar) than new nouns and their antecedents (0.57 versus 0.70, two-sample *t*-test *t* = 8.47, *p* < 0.001).

For the EEG experiment, we added 50 filler items to the final set of 200 experimental items. Three fillers served as practice items (one item corresponding to the New, Old and Partial-Match condition each). The other 47 fillers had the same format as the New condition, which was done to increase the percentage of stories without an anaphor. Roughly 60% of the items in each stimulus list contained an anaphor, while 40% of all items contained a new noun.

We followed previous studies on discourse comprehension (Van Berkum et al., 1999a,b; Nieuwland and Van Berkum, 2006, 2008a) by using a mixed-modality design where the context sentences were spoken and the target sentence was written. We created audio-recordings (44.1 kHz sampling) for the four different story contexts. All recordings were performed by the same native-Dutch, female speaker in a sound-shielded booth. This speaker recorded both context sentences for the old condition. For the other three conditions, only the first context sentence was recorded, which was then paired with the second sentence recorded for the Old condition. Because the speaking rate for the recordings was considered slightly too fast for the experiment, the recordings were lengthened by 15% using the Praat software (Boersma and Weenink, 2013). This yielded a speaker rate that was comfortable for listening without being unnaturally slow (as evaluated by two native speakers of Dutch) and without compromising sound quality.

As there were four conditions, we created four stimulus lists. Each list contained 50 items of each condition and 50 filler items. The lists were created such that they never contained multiple conditions of the same item. Next, the four lists were distributed equally among the participants. For each participant, the items in the list were pseudorandomized, such that there were no consecutive trials of the same condition.

### Procedure

After participants had given written informed consent, they were tested in a sound-shielded booth. They were told that the experiment was about understanding mini stories. They were also told that the last sentence of each mini story was about a specific person or object, and that they had to indicate after each trial whether this person or object had been referred to before (‘old’) or not (‘new’). To discourage participants from using a strategy based on noun repetition alone, and to encourage them to establish co-referential relationships between anaphor and antecedent whenever plausible, we told them that anaphors did not have to be exactly the same as antecedent and could be a different word.

Each trial started with a fixation cross. When participants pressed a button, the two spoken context sentences were presented over loudspeakers located on the desk in front of the participant. Then, 700 ms after the end of the audio recording, the third sentence was presented visually, one word at a time, in black letters (font Lucidia Console, size 20) on the center of a computer screen, which had a light gray background. Each word was presented for 300 ms, with an inter-stimulus-interval of 300 ms. Sentence-final words were presented for 550 ms and followed by a blank screen for 300 ms. Subsequently, the old-new question was presented, which could be answered by a button press (left button for “new,” right button for “old”). Participants were asked to minimize eye blinks and body movements during the word-by-word presentation of the third sentence.

The experiment started with three practice trials, after which the experimental trials would be presented. These were presented in five blocks of 50 items. Participants were allowed to take short breaks between blocks. In total, the experiment lasted approximately 80 min.

### EEG Recording

**FIGURE 1:**
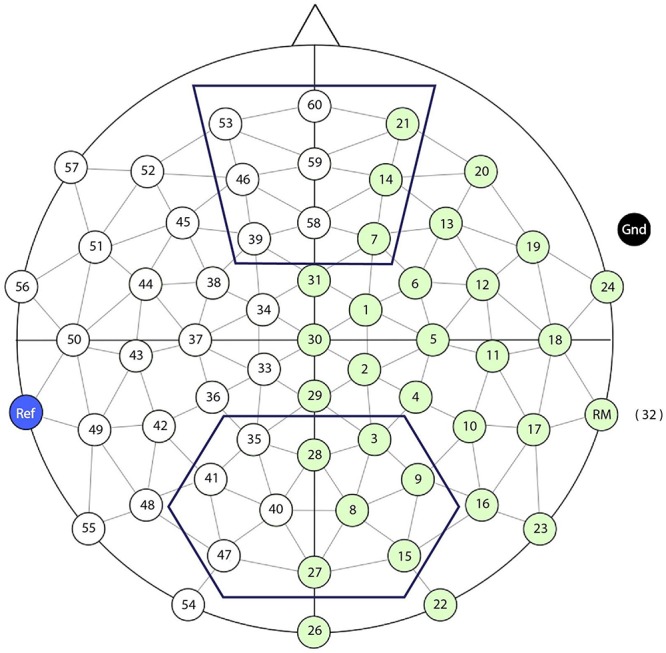
Schematic representation of the 58-electrode array layout. The top and bottom outline represent respectively the Nref and N400/LPC regions of interest.

The electroencephalogram (EEG) was recorded using an MPI custom actiCAP 64-electrode montage (Brain Products, Munich, Germany), of which 58 electrodes were mounted in the electrode cap (see Figure 1). We recorded horizontal EOG with one electrode placed on the outer canthus of the right eye, and vertical EOG with two electrodes placed below both eyes. One electrode was placed on the right mastoid, the reference electrode was placed on the left mastoid, and the ground was placed on the forehead. The EEG signal was amplified through BrainAmp DC amplifiers, referenced online to the left mastoid, sampled at 500 Hz and filtered with a passband of 0.016-249 Hz. Pre-processing was performed in BrainVision Analyzer 2.1 (Brain Products, Munich, Germany).

### ERP Pre-processing and Analysis

We first visually inspected the raw data and interpolated bad channels if they contained strong 50 Hz line noise or indicated broken electrodes. The data was then band-pass filtered at 0.03–40 Hz (24 db/oct) and re-referenced to the average of the left and right mastoid. Segments were extracted ranging from −500 to 1500 ms relative to CW onset, and segments in which an incorrect response had been given (‘new’ response to old, partial-match or ambiguous; ‘old’ response to new) were rejected. Based on visual inspection, we then removed bad segments containing large eye movements, muscle activity, or amplifier blocking. Subsequently, we removed blinks, eye-movements and steady muscle activity using Independent Component Analysis (ICA; Jung et al., 2000), using ICA weights from a 1 Hz high-pass filtered version of the data. We then performed baseline correction using a 250 ms pre-CW baseline interval, and then automatically rejected segments that contained voltage values exceeding ±90 μV. We excluded three participants who retained fewer than 140 trials in total (35 per condition, on average). In the final set of trials for the ERP analysis, participants had on average 45.3 trials for ambiguous nouns, 42.8 for old nouns, 43.7 for new nouns, and 35.4 for partially matching nouns.

For analysis of the behavioral responses, we performed mixed effects logistic regression (Baayen et al., 2008) in the R software (R Core Team, 2018)^9^, with correction for multiple comparisons using the Holm method (Holm, 1979, implemented in the p.adjust function). For the ERP analysis, we performed a linear mixed-effects analysis (Baayen et al., 2008). The ERP analyses were done separately for three dependent variables corresponding to a specific region of interest (ROI): N400, LPC and Nref.

For the N400, we calculated the average voltage across the centroparietal electrodes 35, 28, 3, 41, 40, 8, 9, 47, 27, 15 in a 300–500 ms window after CW onset, for each trial and each participant (see Figure 1). For the LPC, we calculated the average voltage across these same centroparietal electrodes but in a 500–1000 ms window after CW onset. For the Nref, we calculated the average voltage across the frontal electrodes 53, 60, 21, 46, 59, 14, 39, 58, 7 in a 300–1500 ms window after CW onset.

The variable ‘condition’ had four levels: old, ambiguous, new, and partial, which were deviation coded. The models had subject and item as random effects, and initially included a by-subject and by-item random slope for ‘condition’ (Barr et al., 2013) but these slopes were removed due to convergence issues. We compared models with a chi-square test using R’s anova() function, and treated *p*-values below α = 0.05 as statistically significant. For the N400 and LPC, we performed all (Holm-corrected^10^) pairwise comparisons between given anaphors, partially matching anaphor and novel nouns, but not ambiguous anaphors. For the Nref, we specifically tested whether ERPs elicited by ambiguous anaphors were more negative than the mean ERP values across the other three conditions.

### Oscillatory Pre-processing and Analysis

After interpolation of bad channels, we band-pass filtered the data at 0.1–100 Hz (24 db/oct), re-referenced the data to the average of the left and right mastoid, and segmented the data into epochs ranging from −1000 to 2500 ms relative to CW onset. After this, we used the same procedure as for the ERP analysis to reject trials with incorrect responses or artifacts and to perform ICA-based correction for blinks, eye movements and steady muscle activity. The resulting dataset for each participant contained many artifact-free trials with voltage values exceeding ±100 μV. We therefore considered the preregistered ±100 μV amplitude criterion to be too conservative, excluding on average 50.9 trials per participant (*SD* = 38.6). We chose to use a more liberal difference criterion, which excluded segments for which the difference between the maximum and minimum voltage exceeded 200 μV (see Coopmans and Nieuwland, 2019). We excluded four participants who retained fewer than 140 trials in total. In the final set of trials for the time-frequency analysis, participants had on average 46.5 trials for ambiguous nouns, 45.2 for old nouns, 43.2 for new nouns, and 37 for partially matching nouns.

Time-frequency analysis was performed using the Fieldtrip toolbox (Oostenveld et al., 2011). We performed time-frequency analysis in two different, but partially overlapping frequency ranges. For the low (2–30 Hz) range, we used a 400-ms Hanning window to compute power changes in frequency steps of 1 Hz and time steps of 10 ms. For the high (25–90 Hz) frequency range, we computed power changes with a multitaper approach (Mitra and Pesaran, 1999) based on Slepian sequences as tapers, with a 400-ms time-smoothing and a ±5 Hz spectral-smoothing window, in frequency steps of 2.5 Hz and time steps of 10 ms. Then, for each trial, we computed power in the post-stimulus interval as a relative change from a baseline interval spanning from −500 to −250 ms relative to CW onset. Average power changes per subject were computed for each condition separately.

For the statistical analysis, we pre-registered three ROIs: theta (4–7 Hz) activity in the 0–1000 ms interval after critical word onset, averaged over frequency but not over time; low gamma (35–45 Hz) in the 400–600 ms interval, average over both frequency and time; high gamma (60–80 Hz) in the 500-1000 ms interval, average over both frequency and time. In addition to these ROIs, we also pre-registered an analysis of the 200–1500 ms time window that did not average activity over time or frequency.

We used cluster-based random permutation tests (Maris and Oostenveld, 2007) to compare differences in oscillatory power across conditions. In brief, this statistical test works as follows: first, by means of a two-sided dependent samples t-test we performed all pairwise comparisons between the four conditions on the three dependent variables described above, which yielded uncorrected *p*-values. Neighboring data triplets of electrode, time and frequency-band that exceeded a critical α-level of 0.05 were clustered. Clusters of activity were evaluated by comparing their cluster-level test statistic (sum of individual *t*-values) to a permutation distribution that was created by computing the largest cluster-level *t*-value on 1000 permutations of the same dataset. Clusters falling in the highest or lowest 2.5th percentile were considered statistically significant. We used the correct-tail option that corrects *p*-values for doing a two-sided test, which allowed us to evaluate *p*-values at α = 0.05.

## Results

### Old/New Judgments

**FIGURE 2:**
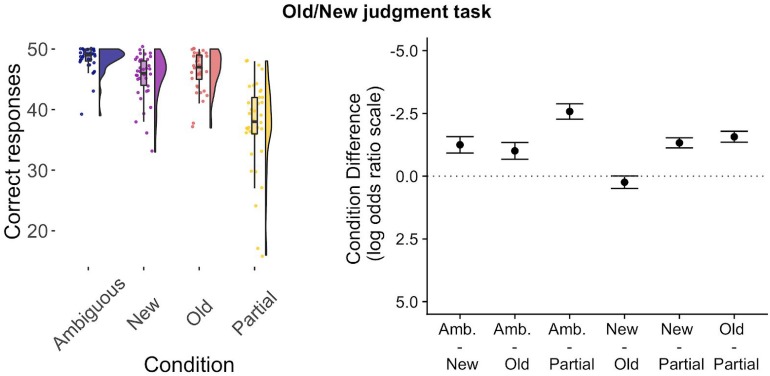
Results from the Old/New judgment task from the subjects included in the ERP analysis. The left graph shows the behavioral accuracy per condition, plotted as the number of correct responses using raincloud plots (Allen et al., 2019), with each point representing a single participant, including the corresponding density and box plot. The right graph shows all pairwise differences between conditions, plotted as the estimated marginal means difference with the 95% confidence level.

Participants responded most accurately to ambiguous nouns, then to old nouns, new nouns and partially matching nouns (Figure 2; this figure and the analysis only includes participants used in the ERP analysis, average number of trials per conditions is *M* = 48.3, 46.3, 45.4, and 37.5, respectively). Our analysis revealed a strong effect of condition (χ^2^ = 517.06, *p* < 0.001) and differences between all pairs of conditions, with the strongest effects seen in comparison to the partially matching condition.

### Pre-registered ERP Analyses

#### N400 (300–500 ms)

**FIGURE 3:**
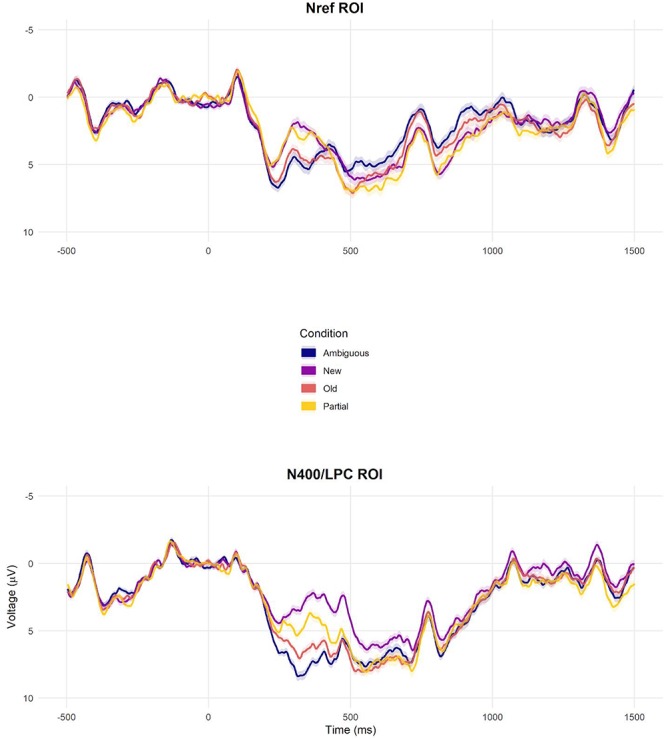
Grand-average ERPs per condition at the frontal Nref ROI and the central-posterior N400/LPC ROI, reflecting average activity from the channels within each ROI. In these and subsequent ERP plots, negative voltage is plotted upwards. Color-shaded areas show the within-subject standard error of the condition mean per time sample (parametric ‘Cousineau–Morey’ confidence intervals, see Cousineau, 2005; Morey, 2008).

**FIGURE 4:**
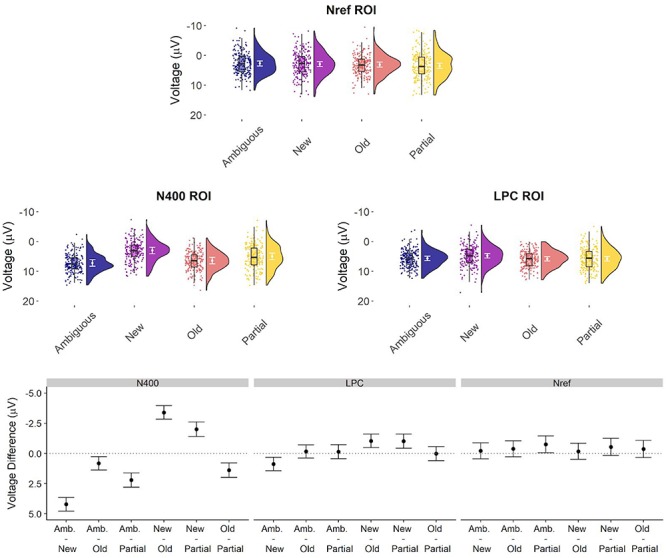
Voltage per condition in the pre-registered ROIs and pairwise comparisons. **Upper graphs:** observations for each condition at the three ROIs using raincloud plots (Allen et al., 2019), with each point representing mean voltage associated with one item (averaged over subjects), the corresponding density and box plot, and the estimated marginal means per condition with the 95% confidence level (plotted in white on top of the density plot). **Lower graphs:** pairwise contrasts for each ROI with 95% confidence level.

Our experimental manipulation was associated with modulations of activity in the N400 region of interest (χ^2^ = 196.18, *p* < 0.001), with most negative amplitude elicited by new nouns, followed by partially matching, old and ambiguous nouns in that order (Figure 3; ERP waveforms at all individual channels are shown in Supplementary Figure 1). Pairwise follow-up tests revealed reliable differences between all conditions (Figure 4).

#### LPC (500–1000 ms)

Our experimental manipulation was also associated with modulations of activity in the subsequent LPC time window (χ^2^ = 13.311, *p* = 0.004; Figures 3, 4 and Supplementary Figure 1). This effect mostly reflected a carry-over effect from the enhanced N400 to new nouns, as the pairwise follow-ups showed that while new nouns elicited reliably more negative voltage than the other three conditions (although for partially matching nouns, this difference was not statistically significant after multiple comparisons correction), these other conditions did not reliably differ from each other.

#### Nref (300–1500 ms)

At the frontal ROI, ambiguous nouns elicit more negative voltage compared to the other conditions (*M* = −0.32, *S.E*. = 0.28; Figures 3, 4 and Supplementary Figure 1), compatible with an Nref effect, but this contrast did not reach the conventional alpha = 0.05 criterion (χ^2^ = 1.3, *p* = 0.25).

### Exploratory ERP Analyses

Our pre-registered ERP analyses showed that EEG activity was most sensitive to whether or not the critical noun had featured in the spoken story context, but did not differentiate anaphoric nouns and new nouns. Although amplitude in the N400 ROI differentiated between all four conditions, this pattern could merely reflect the relative ease of accessing the meaning of a noun that is more strongly related to context words, in other words, it need not reflect the process of anaphor resolution. Moreover, the smallest N400 was obtained for the ambiguous condition, wherein anaphor resolution was not straightforward. Likewise, we did not obtain a clear pattern of correlation between anaphor resolution and modulation of the LPC in the pre-registered ROI. We offer further discussion of these results in the Section “Discussion.”

We considered the possibility that our participants used a strategy whereby they based their initial interpretation on whether the noun had been heard before (old/ambiguous versus new/partial), and subsequently changed this initial interpretation if the new noun could plausibly refer back to an antecedent (partial versus new). Such a strategy could be associated with an ERP effect of partial-matching nouns in a different ROI than the one we pre-registered. We tested for such an effect in two exploratory ERP analyses.

**FIGURE 5:**
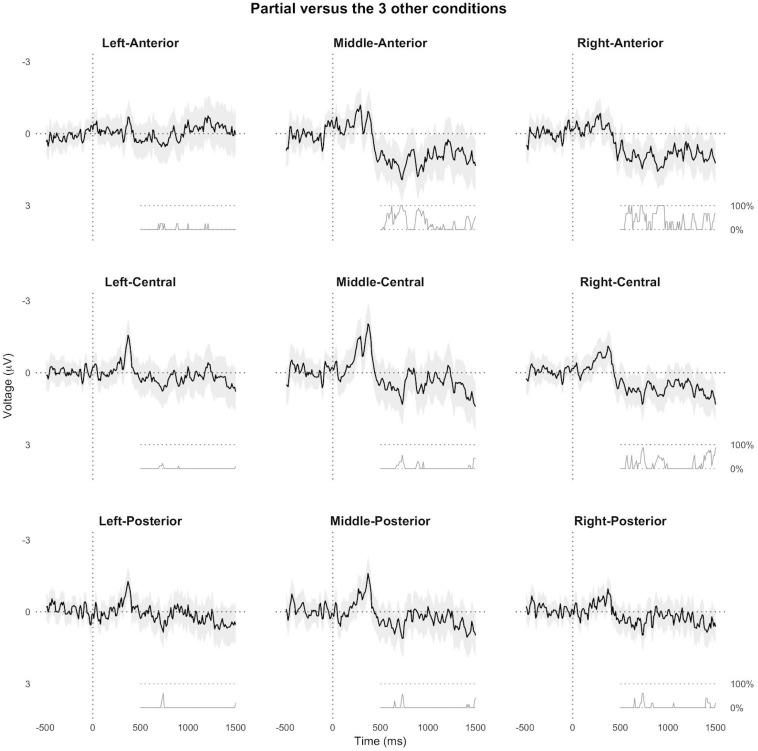
Results of the exploratory mass regression analysis. The black line and gray area correspond to the estimate and 95% confidence interval, respectively, of the contrast partial-matching versus the mean of the other three conditions, plotted at each timepoint in 9 ROIs. Below each ERP, we plot the percentage of channels per ROI showing a statistically significant difference after multiple comparison correction, in the 500–1500 ms time window after noun onset.

Our first exploratory analysis employed a mass regression approach (e.g., Groppe et al., 2011; Nieuwland et al., 2019) to test for later effects in the data segments from the pre-registered analysis. We down-sampled the data to 100 Hz and then ran a mixed-effects model analysis to test the contrast partial-match against the mean of the other three conditions at each electrode channel and at each data point between −500 ms before to 1500 ms after noun onset. This yielded an effect estimate and standard error for each timepoint and channel. The associated *p*-values in the post-N400 window (from 500 to 1500 ms after noun onset) were corrected for multiple comparisons using the Benjamini and Hochberg method to control the false discovery rate (Benjamini and Hochberg, 1995). The resulting estimates are plotted as ERPs along with the corrected *p*-values (Figure 5 for an ROI-based plot, and Supplementary Figure 2 for a plot of activity at all individual channels and highlighting of statistically significant samples after multiple comparison correction), revealing that partially matching nouns elicited more positive voltage than the other three conditions, particularly at middle-frontal, right-frontal and right-central channels in the post-N400 time window. Of note, the ROIs in Figure 5 contain different numbers of channels.

**FIGURE 6:**
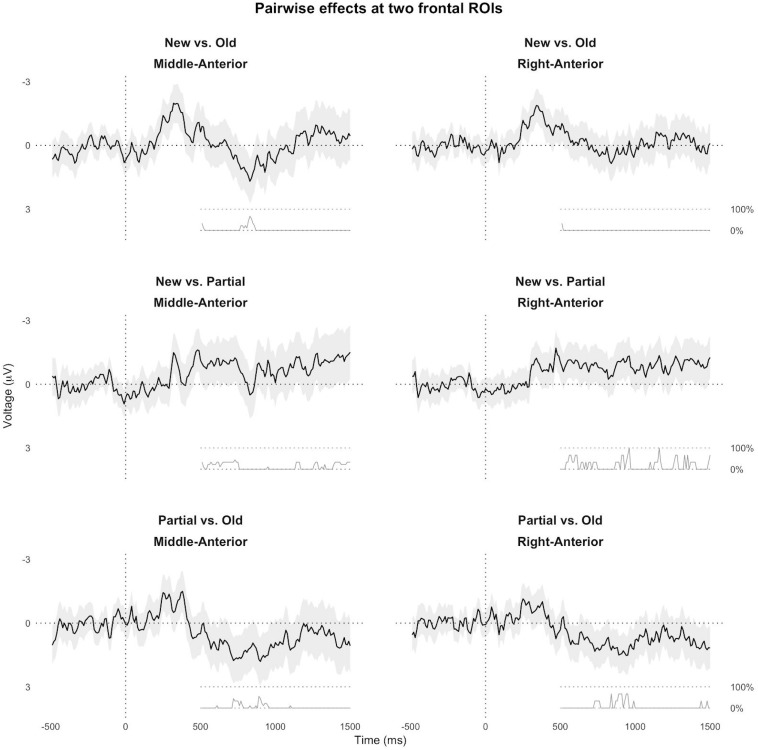
Results of the exploratory mass regression analysis. The black line and gray area correspond to the estimate and 95% confidence interval, respectively, of three pairwise contrasts, plotted at each timepoint in a middle- and right-anterior ROI.

We performed similar analyses that directly compared partially matching nouns to only new or old nouns, and new nouns to old nouns (Figure 6 and Supplementary Figures 3–5). These results suggest that the processing consequences of the partial match condition extended beyond the pre-registered ROI, and that partially matching nouns and new nouns both elicited a frontal positive ERP effect compared to old nouns in the post-N400 window around 500–1000 ms.

**FIGURE 7:**
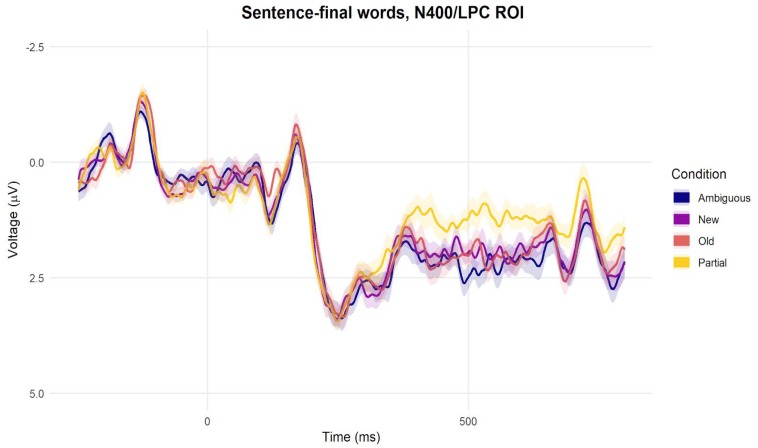
Grand-average ERPs per condition elicited by sentence-final words at the central-posterior N400/LPC ROI.

Our second exploratory analysis involved activity elicited by sentence-final words, to which we applied the same pre-processing steps as to the critical nouns (except that we segmented epochs of shorter duration, until 800 ms after word onset). As shown in Figure 7 (and corresponding Supplementary Figure 6 showing ERP waveforms at all individual channels), partially matching nouns elicited more negative voltage than the other conditions. Using the N400/LPC spatial ROI, a contrast-based analysis showed more negative voltage for the partially matching nouns when compared to the mean of voltage for the other nouns (*M* = −0.48, *S.E.* = 0.24, *t* = 2.01, *p* = 0.044). This pattern is compatible with a sentence-final N400 effect, which extended beyond 500 ms after word onset (see also Nieuwland, 2014). In sum, both our exploratory analyses suggested enhanced processing difficulty associated with partial-matching nouns that extended up to the end of the sentence.

### Pre-registered Time-Frequency Analyses

**FIGURE 8:**
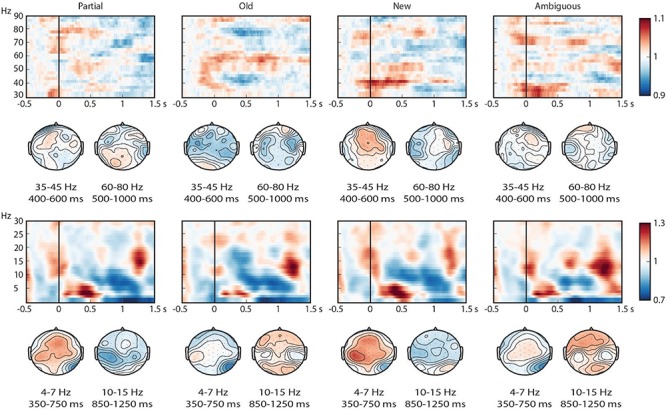
Time-frequency representations of all conditions at selected electrodes (centro-frontal electrode 59 for the low frequency range (2–30) and electrode 58 for the high (30–90) frequency range). Power is represented as a relative change from activity in the baseline interval. Topographical plots are presented for theta, beta, low gamma and high gamma.

As shown in Figure 8, all the conditions elicited a visually salient, relative power increase in the theta band in the first 500 ms after noun onset, and a subsequent power decrease in the beta (10–15 Hz) band that extended until approximately 1300 ms after nouns onset. Patterns in the high frequency range were less pronounced.

**FIGURE 9:**
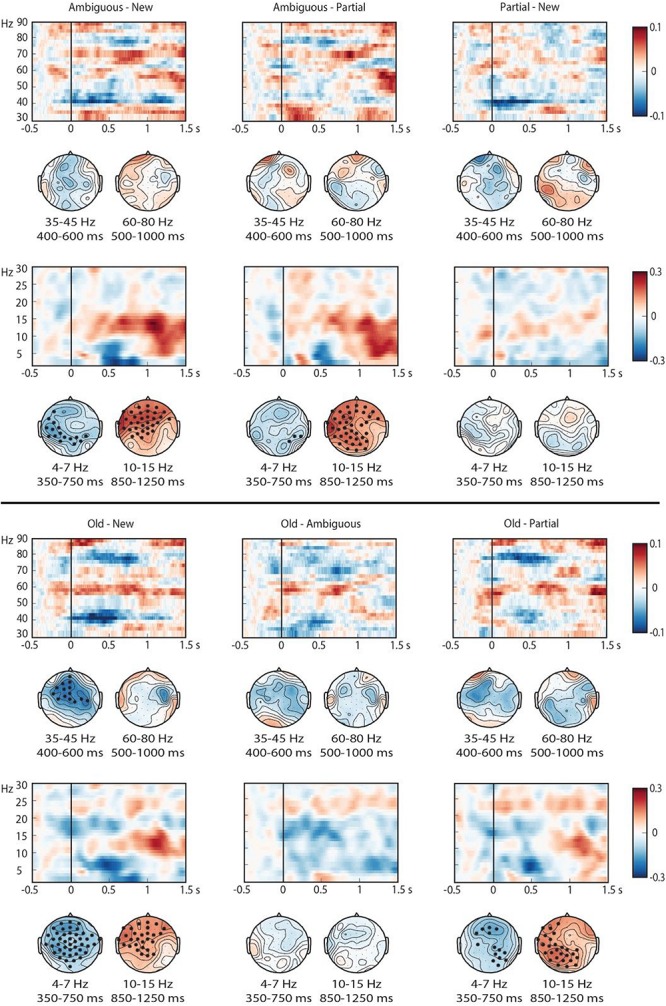
Time-frequency representations of all pairwise contrasts at selected electrodes (centro-frontal electrode 59 for the low frequency range (2–30) and electrode 58 for the high (30–90) frequency range). Power is represented as a relative change from activity in the baseline interval. Scalp topographies for low and high gamma represent the activity within the preregistered time windows, and those for theta and beta reflect the time windows in which effects were most pronounced. Electrodes with significant differences in more than 60% of the attested time points are indicated with an ^∗^.

As shown in Figure 9, the pairwise contrasts showed activity differences in the pre-registered ROIs but also in the beta range. In the theta (4–7 Hz) ROI, the contrasts Old-New, Old-Partial, Ambiguous-New, and Ambiguous-Partial showed significant differences (Table 2): new and partially matching nouns elicited greater theta power increases than old and ambiguous nouns. Ambiguous nouns also elicited greater theta power than old nouns, suggested by a smaller yet sizeable cluster, although this contrast did not reach the alpha = 0.05 criterion. The results suggested no clear difference between partially matching and new nouns.

**TABLE 2:** Time-frequency effects in the theta range (4–7 Hz) occurring in the 0–1000 ms time window after noun onset.

	**Cluster *t*-value**	**Cluster size**	***p*-value**
Old – New	−6002	1823	0.002/0.012
Old – Partial	−3127	1112	0.008/0.032
Old – Ambiguous	−1287	506	0.066/0.132
Ambiguous – New	−3562	1150	0.006/0.030
Ambiguous – Partial	−2552	877	0.010/0.032
Partial – New	−108	45	0.745/0.745

*In this and all following tables, the values correspond to the largest cluster that was found for each comparison. We report uncorrected/corrected *p*-values for each pairwise comparison. In this and following tables, for each test df = 34.*

In the low gamma (35–45 Hz) ROI, new nouns elicited greater power than old nouns in the 400–600 ms time window after critical word onset (Table 3). Partially matching and ambiguous nouns also elicited greater low gamma power than old nouns, although these clusters did not reach the α = 0.05 threshold.

**TABLE 3:** Time-frequency effects in the lower gamma range (35–45 Hz) occurring in the 400–600 ms time window after noun onset.

	**Cluster *t*-value**	**Cluster size**	***p*-value**
Old – New	−846	347	0.038/0.228
Old – Partial	−332	135	0.090/0.370
Old – Ambiguous	−354	149	0.074/0.370
Ambiguous – New	No cluster	No cluster	No cluster
Ambiguous – Partial	No cluster	No cluster	No cluster
Partial – New	No cluster	No cluster	No cluster

In the high gamma (60–80 Hz) ROI, there were no significant differences in the 500–1000 ms time interval after critical word onset (Table 4), although a sizeable cluster that did not reach the conventional threshold suggested more power for partially matching nouns compared to old nouns.

**TABLE 4:** Time-frequency effects in the higher gamma range (60–80 Hz) occurring in the 500–1000 ms time window after noun onset.

	**Cluster *t*-value**	**Cluster size**	***p*-value**
Old – New	−144	56	0.302/1.00
Old – Partial	−697	271	0.070/0.42
Old – Ambiguous	No cluster	No cluster	No cluster
Ambiguous – New	60	27	0.356/1.00
Ambiguous – Partial	No cluster	No cluster	No cluster
Partial – New	No cluster	No cluster	No cluster

Our pre-registration also included additional analyses of a more exploratory nature that tested for effects in the 200–1500 ms time window after noun onset without averaging over time or frequency, for lower (2–30 Hz) and higher (30–90 Hz) frequencies separately. This analysis revealed six significant clusters (Supplementary Table 1), all of which were in the low (2–30 Hz) frequencies. However, some of the effects in this analysis were composed of seemingly unrelated clusters. For this reason, based on visual inspection, we performed an extra (exploratory) analysis which averaged over the beta (10–15 Hz) frequency range within the 0–1500 ms time window after critical word onset.

This analysis revealed four clusters with greater power for old and ambiguous nouns compared to new and partially matching nouns (Table 5). Visual inspection (Figure 9) indicates that these clusters were most prominent around 1000 ms after noun onset.

**TABLE 5:** Time-frequency effects in the 10–15 Hz time-frequency analysis of the 0–1500 ms time window after critical noun onset.

	**Cluster *t*-value**	**Cluster size**	***p*-value**
Old – New	3955	1451	0.010/0.032
Old – Partial	5032	1857	0.008/0.032
Old – Ambiguous	−1886	730	0.056/0.112
Ambiguous – New	1077	3751	0.002/0.012
Ambiguous – Partial	8974	3095	0.004/0.020
Partial – New	−247	97	0.599/0.599

### Exploratory Time-Frequency Analyses

**FIGURE 10:**
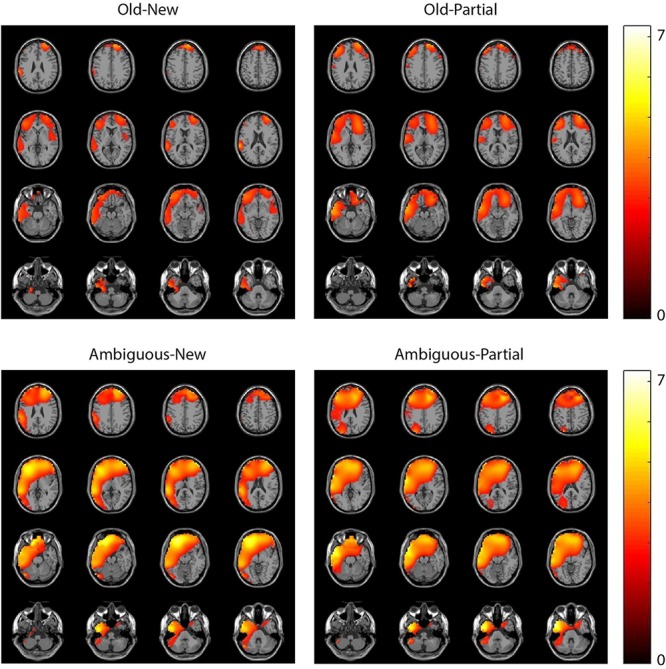
Beamformer source localization of the pair-wise beta effects (10–15 Hz). Colorbar represents *t*-values, masked for significance.

We performed two types of exploratory analysis. First, we tried to localize the sources of the obtained time-frequency effects using beamformer analysis (Groß et al., 2001; for a detailed description of the method as applied to similar data sets, see Nieuwland and Martin, 2017; Coopmans and Nieuwland, 2019^11^). For the theta effects, which were focused on the 350–850 ms interval after critical word onset, this analysis did not reveal any statistically significant clusters. For the beta effects, the analysis was focused on a 700–1200 ms time window after critical word onset. This suggested a distributed source ranging from (pre)frontal to temporal areas (see Figure 10), with a slight left hemispherical focus.

**FIGURE 11:**
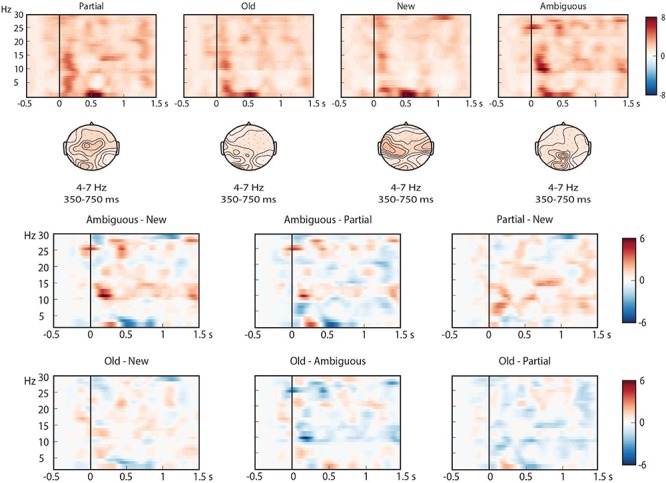
Time-frequency representations (2–30 Hz) of the phase-locked activity elicited by the critical word. The top graphs show the results for individual conditions, based on centro-frontal electrode 59. Power is represented as a relative change from activity in the baseline interval. Topographical plots represent 4–7 Hz theta activity in a 350–750 time window. The bottom graphs show the corresponding pairwise contrasts.

To ensure that the reported time-frequency effects in the 2–30 Hz frequency band provide information over and above the information found in the ERPs, we performed a second exploratory analysis. Similar to Bastiaansen et al. (2008), we tested whether the reported time-frequency effects could also be obtained from phase-locked activity alone by performing the same analysis on averaged ERPs per condition per subject (see Cohen, 2014, for limitations of this method). When a cluster is present in our pre-registered analysis, but absent in this phase-locked time-frequency analysis, we have greater evidence that the observed effects are independent of the ERP effects. We found two 4–7 Hz theta-band effects (Figure 11), one in the Old-New contrast (*p* = 0.016), and one in the Ambiguous-New contrast (*p* = 0.036). Both of these clusters are in the same negative direction and in roughly the same time windows (around 400 ms after critical word onset) as the pre-registered theta effects. This means that for these contrasts, part of our effect in the theta-band is phase-locked. However, visual inspection of the time-frequency representations (Figures 8, 11) leads us to believe that not everything in the pre-registered theta cluster can be explained by the phase-locked information alone (i.e., the pre-registered theta clusters cover higher frequencies). The fact that the phase-locked effects are only present in 2 out of the 4 contrasts in which we found a significant cluster in the pre-registered analysis corroborates this line of reasoning.

## Discussion

In this EEG study, we used ERP and time-frequency analyses to investigate the resolution of anaphoric noun phrases during discourse comprehension. We had a particular interest in how people resolve anaphors that are semantically related but different in form from the antecedent (e.g., *martian-alien*). Participants listened to story contexts that described two antecedents, and subsequently read a target sentence with a critical noun phrase. Depending on the story context, the critical noun phrase lexically matched one antecedent (‘old’), matched two antecedents (‘ambiguous’), partially matched one antecedent in terms of semantic features (‘partial-match’), or introduced another referent (non-anaphoric, ‘new’). After each story, participants judged whether the noun referred back to an antecedent (an ‘old/new’ judgment), and we used these responses to select trials in which participants arrived at the ‘intended’ interpretation (‘old’/anaphoric for old, ambiguous and partially matching nouns, ‘new’/non-anaphoric for new nouns) for further analyses.

Pre-registered ERP analyses revealed modulation of the N400 ERP component by the status of the critical noun. We observed a stepwise decrease (becoming less negative) in N400 amplitude: the new condition had the highest N400 amplitude, then partially matching, old and finally, ambiguous nouns showed the lowest amplitude. We take this to reflect the context-based facilitation of access to the semantic meaning of the noun (e.g., Kutas and Federmeier, 2000; Burkhardt, 2006, 2007; Nieuwland and Van Berkum, 2008a; Lau et al., 2009). In addition, although we did not find an Nref effect that was statistically significant at the conventional α = 0.05 threshold, ambiguous nouns did elicit a sustained, frontal negativity compared to the other nouns, which is compatible with previous effects of referential processing difficulty (Van Berkum et al., 1999a, 2003; Nieuwland et al., 2007). Finally, additional exploratory ERP analyses revealed that partially matching nouns and new nouns had similar positive ERP components in the early part of the post-N400 window, but that they diverged later on in the sentence and in response to sentence-final words.

Pre-registered time-frequency analyses were performed in theta, low gamma and high gamma ROIs. Theta effects were most pronounced and sensitive to whether or not the noun had been heard in the context, and did not differentiate partially matching nouns and new nouns. These theta effects could not entirely be explained as a time-frequency effect of the phase-locked ERP effects (see also Bastiaansen et al., 2005, 2008). Gamma effects were weak but suggested a power decrease for old nouns in the lower gamma frequency band (35–45 Hz). Exploratory time-frequency analyses further revealed strong differences between conditions in the beta (10–15 Hz) frequency range, primarily demonstrating sensitivity to whether or not the noun had occurred before. The time-frequency patterns therefore did not reveal a clear difference between partially matching and new nouns, as would be indicative of anaphor resolution.

The combination of our behavioral, ERP and time-frequency results suggests the cognitively demanding nature of resolving the anaphoric meaning of partially matching nouns. In the sections below, we will unpack this conclusion for both ERP and time-frequency results separately.

### Interpretation of ERP Results

Our N400 results suggest that the semantic meaning of partially matching nouns was easier to access than that of new nouns, but harder to access than that of old or ambiguous nouns. Nevertheless, three distinct results in the later time windows suggest that the referential, anaphoric meaning of partially matching nouns may have been difficult to establish. Firstly, in approximately the 500–1000 ms time window after noun onset, partially matching nouns and new nouns both elicited enhanced positivity compared to ambiguous and old nouns at the frontal channels (Figure 6 and Supplementary Figures 3, 4), suggesting that partially matching nouns may have been initially considered as new, non-anaphoric nouns^12^ (e.g., Burkhardt, 2006, 2007; Brouwer et al., 2012; Wang and Schumacher, 2013). Secondly, in an even later time window, approximately 1000–1500 ms, partially matching nouns elicited more positive voltage compared to old nouns and new nouns, while new nouns elicited more negative voltage than old nouns (Figure 6 and Supplementary Figures 3–5). This late window thus revealed processing difficulty associated with partially matching nouns and with new nouns, but each with a distinct ERP profile (and thus presumably a distinct processing mechanism). Finally, ERPs elicited by sentence-final words suggested downstream processing difficulty for partially matching nouns compared to the other conditions (Figure 7 and Supplementary Figure 6).

We think that the processing difficulty associated with partially matching nouns stems from the combination of the materials and the task. The old/new task might have focused the participant’s attention on the lexical form of the words, rather than their referential meaning. For partially matching nouns, participants were required to remember two lexically different antecedents over the course of two spoken sentences, and then establish an anaphoric interpretation on yet another different word. Although the partially matching nouns were related in meaning to and sometimes synonymous with one antecedent, such anaphors (even the synonyms) may have been difficult to immediately recognize as such, especially in an experimental setting where the target noun on many trials introduced a new referent and where the task could have implied focus on lexical form. In comparison, the three other conditions were easier in terms of task demands. For ambiguous and old nouns, the task could be performed based on lexical repetition alone, and for ambiguous nouns participants only needed to remember one antecedent. The latter seemed to matter for the task, as participants were more accurate in recognizing ambiguous nouns than old nouns. For new nouns, participants only needed to remember one antecedent, and they could often rely on coarse semantic cues that ruled out an anaphoric interpretation, such as animacy or biological gender, or on semantic role information (e.g., patient–doctor).

Several patterns in our results suggest that although participants did ultimately establish the anaphoric meaning of partially matching nouns, they may have initially treated them as new, perhaps as part of a strategy that focused first on identifying lexical repetition and subsequently resolving the anaphor based on meaning. For example, new and partially matching nouns elicited a similar frontal, post-N400 positive effect compared to old nouns. This effect could be linked to the introduction of a new referent (discourse updating; Burkhardt, 2006), but, alternatively, may simply be due the unexpectedness of these nouns. Likewise, as discussed in the next section, the time-frequency results did not reveal clear differences between partially matching and new nouns. If participants switched from a non-anaphoric to an anaphoric interpretation (from ‘new’ to ‘old’) later on in the sentence, this could have caused difficulty keeping up with the remainder of the unfolding sentence. Compatible with this idea, sentence-final words following partially matching nouns elicit an N400-like effect compared to the other three conditions. Several studies have reported N400-like negativities for sentence-final words of unexpected or otherwise difficult sentences (Anderson and Holcomb, 2005; Paczynski and Kuperberg, 2012; Nieuwland, 2014; Vega-Mendoza et al., 2018), suggestive of continued sentence comprehension difficulty. Such effects may be more pronounced when participants perform a meta-linguistic judgment task (Nieuwland, 2014; Vega-Mendoza et al., 2018).

We emphasize that although participants in our experiments may have found it cognitively demanding to resolve partially matching anaphors, it is unclear whether this generalizes to regular language settings, where preceding discourse and surrounding visual context often facilitate anaphor resolution, or to a situation where the context only contains a single antecedent (for discussion, see Dell et al., 1983; O’Brien et al., 1986). Likewise, it is possible that without the explicit task in our experiment to create anaphoric relations, participants would arrive at a non-anaphoric interpretation for partially matching nouns more often or even most of the time (see also O’Brien et al., 1997; Levine et al., 2000; Klin et al., 2004; Klin et al., 2006).

One further aspect of our ERP results is noteworthy, namely that while ambiguous nouns did not elicit robust Nref effects, they elicited less negative voltage in the N400 ROI compared to old nouns. The latter pattern may be caused by the noun repetition in the story context, because two identical context nouns may lead to a stronger repetition priming effect than a single noun (Van Petten et al., 1991). Previous studies did not observe such an effect, perhaps because they did not use identical context nouns (e.g., Van Berkum et al., 1999a, 2003; Nieuwland et al., 2007; Nieuwland and Van Berkum, 2008a), but instead used constructions such as “one alien who…and another one who.” Moreover, as noted earlier, remembering one antecedent was easier than two, as suggested by the recognition task results^13^.

In sum, our ERP analyses generated a varied range of effects. While our results showed relatively clear effects associated with referent activation, they are somewhat inconclusive in the sense that we could not conclusively tie any single effect specifically to the difference between old or new referents (discourse updating). This may have had to with the task demands of our experiment, and with the fact that old and partially matching anaphors showed little similarity in brain responses despite being both interpreted as anaphoric.

### Time-Frequency Results

Whereas the ERP results clearly differentiated old from ambiguous nouns, and partially matching from new nouns, the time-frequency results primarily yielded effects of lexical repetition: effects of old/ambiguous versus new/partially matching, with some evidence for a difference between old and ambiguous nouns (which differed in number of repetitions), but no clear difference between new and partially matching nouns (which were both lexically new and thus did not differ in repetition). The observed effects were strong in the theta and beta frequency range, but much less so in the gamma frequency range. The time-frequency analysis alone therefore did not allow us to identify activity that might be related to resolution of partially matching nouns, and this suggests that ERPs are more sensitive to these processes. However, we emphasize that time-frequency analysis typically requires a larger number of trials than ERP analysis to obtain stable estimates (e.g., Bastiaansen et al., 2013). Our data contained relatively low trial numbers in particular for partially matching nouns, which received the lowest number of correct ‘old’ responses. This will have decreased our ability to pick up on relevant differences.

We found greater theta (and, to a lesser extent, gamma) power for new/partially matching nouns than for old/ambiguous nouns. These patterns clearly differ in their directionality and functionality from recent findings on proper name anaphors (Coopmans and Nieuwland, 2019), which revealed increased theta (and to a lesser extent, low gamma) for old/repeated compared to new proper names. The theta effects in these studies also differ in the frequency range they appear to cover. It is possible that these differences somehow stem from the differences in anaphor type, in particular because proper names (of unfamiliar discourse characters) contain much less semantic content than noun phrases.

One possibility is that theta power correlates with the amount of semantic information that is retrieved from long-term memory (e.g., Bastiaansen et al., 2005, 2008). In Coopmans and Nieuwland (2019), this would not differ between old and new proper names, perhaps because the names themselves contain little semantic content. For new noun phrases in the current study, however, the full meaning of the word will be retrieved, whereas for old noun phrases most of the relevant meaning may already be active due to the first presentation. Another difference was that the stimuli used by Coopmans and Nieuwland were all written, whereas the current study combined spoken with written language. It is possible that theta effects are sensitive not only to lexical repetition but also to repetition of form. Beyond these differences in anaphor type and modality, other differences in terms of task demands may be relevant too. For example, participants in our experiment may have focused strongly on word repetition to perform the task, at the expense of attention to the meaning of the unfolding story. Our time-frequency effects may thus be related to repetition priming effects (e.g., Gruber and Müller, 2004), which could explain why we also obtained power differences between old and ambiguous nouns (which differed in number of repetitions). At any rate, our results demonstrate that theta and gamma effects do not depend on anaphoricity alone. This might make their use to study anaphor comprehension less straightforward than previously suggested (Nieuwland and Martin, 2017; Coopmans and Nieuwland, 2019), although it remains unclear to what extent the observed theta/gamma effects are driven by the task demands. A dedicated follow-up study could shed light on this issue by directly comparing repetition/anaphoricity effects for proper names and noun phrases, or, for instance, by directly manipulating the semantic distance of old and new nouns.

While the effects in the theta frequency band were relatively strong, effects in the gamma range were very weak and inconclusive. One explanation for this lack of results is that there is relatively lower power in the gamma band compared to lower frequency bands, which may make it rather hard to obtain clear gamma effects with a low number of trials, as in the current study. Another explanation could be that gamma activity is primarily sensitive to sentence/discourse-level semantic integration costs (e.g., Peña and Melloni, 2012; Rommers et al., 2013; Fedorenko et al., 2016; Nieuwland and Martin, 2017; Coopmans and Nieuwland, 2019), which was not manipulated in our experiment (in contrast to, for example, a comparison between semantically incongruent and congruent words, see Coopmans and Nieuwland, 2019).

In addition to the effects in the pre-registered theta and gamma ROIs, we found greater beta (∼10–15 Hz) power for old/ambiguous nouns than for new/partially matching nouns, and to some extent for ambiguous nouns compared to old nouns. Beamformer source localization suggested a fairly widely distributed, prefrontal/temporal source with a left hemisphere bias. Beta effects have previously been observed in a wide range of language comprehension studies (for a review, see Weiss and Mueller, 2012; Lewis et al., 2016). One proposal is that beta power is related to maintenance/changes in the current mode of processing and representation of a sentence-level meaning (Lewis et al., 2016), which is based on observed decreases in beta power to unexpected stimuli (e.g., Engel and Fries, 2010). Our results seem compatible with this proposal. Another proposal is that beta synchronization serves to bind distributed sets of neurons into a coherent representation of (memorized) contents during language processing (Weiss and Mueller, 2012).

We refrain from claims about the functional significance of these unanticipated effects. Moreover, we emphasize the fact that, in terms of condition-wise patterns, beta power behaved in largely the same way as theta power, which complicates a functional differentiation of these frequency bands. None of the frequency bands clearly differentiated new from partially matching nouns and could therefore be linked to the difference between anaphoric and non-anaphoric meaning, and all of the frequency bands showed some sensitivity to the difference between old and ambiguous names, suggesting sensitivity to either lexical repetition or to the task demands. What does differ between the frequency bands, however, is the directionality of the effects (increased beta power but decreased theta/gamma power for repeated nouns compared to non-repeated nouns; see Lundqvist et al., 2011, for a similar distinction between these frequency bands in relation to working memory load), the timing of the effects (theta and gamma effects occurred within roughly the first 1000 ms after noun onset, beta effects occurred later), and possibly the underlying neural source of these effects.

In sum, as with the ERP results, our time-frequency results did not allow us to tie one specific effect to anaphoric meaning, and they were chiefly driven by noun repetition. We suspect that the task demands of our experiment were the main driving force behind these effects.

## Conclusion

The flexible nature of human language allows people to establish referential relationships between words that differ in meaning. Very little work to date has examined the neural processes that may underlie such anaphoric interpretations. We addressed this issue in an EEG study on discourse comprehension, wherein we investigated the ERP and time-frequency correlates of how people resolve noun phrases, and in particular how they resolve anaphoric nouns that either lexically match or mismatch the intended antecedent. The N400 ERP component demonstrated initial sensitivity to noun repetition and semantic overlap, corresponding to repetition and semantic priming effects, respectively. A subsequent frontal positivity demonstrated sensitivity to whether the noun had been repeated, suggesting that partially matching anaphors may have been processed as new nouns temporarily. ERPs in even later time windows and ERPs time-locked to sentence-final words suggested that partially matching nouns and new nouns had different effects on comprehension. In contrast to the ERP results, the time-frequency results primarily demonstrated sensitivity to noun repetition, and did not differentiate partially matching anaphors from new nouns. In sum, our results show the ERP and time-frequency effects of referent repetition during discourse comprehension, and demonstrate the potentially demanding nature of establishing the anaphoric meaning of a novel noun.

## Data Availability Statement

In accordance with the Peer Reviewers’ Openness Initiative (https://opennessinitiative.org, Morey et al., 2016), all materials (data, materials, scripts, figures, and supplementary figures) associated with this manuscript are available on https://osf.io/uak8g/.

## Ethics Statement

The studies involving human participants were reviewed and approved by Ethics Committee for Behavioural Research of the Social Sciences Faculty at Radboud University Nijmegen. The patients/participants provided their written informed consent to participate in this study.

## Author Contributions

MN designed the experiment and wrote the manuscript. CC and RS collected the data and provided crucial edits. All authors analyzed the data.

## Conflict of Interest

The authors declare that the research was conducted in the absence of any commercial or financial relationships that could be construed as a potential conflict of interest.

## Footnotes

1 Partially matching anaphors are particularly taxing to comprehension when they are semantically more specific than the antecedent, like ‘the martian’ referring back to an alien, or when they are atypical of a semantic category rather than typical (e.g., Almor, 1999; Van Gompel et al., 2004).

2 In studies on recognition memory, correctly recognized items are associated with enhanced parietal LPC responses compared to correctly rejected items, which is referred to as the parietal old/new LPC effect (e.g., Van Petten and Senkfor, 1996; Rugg and Curran, 2007; Voss and Paller, 2009). It is unknown whether such LPC effects are related to LPC effects associated with anaphoric processing.

3 The brain continuously generates neural oscillations at a wide range of frequencies and the phase of these frequencies may differ at stimulus onset. By averaging over trials, ERP analysis cancels out activity that differs in phase over trials. However, a stimulus may impact the activity in a specific frequency band without changing its phase (e.g., Bastiaansen et al., 2013; Lewis et al., 2015). This impact cannot be detected in an ERP analysis, but can be detected in time-frequency analysis of spectral power.

4 Boudewyn et al. (2015) investigated correlations between antecedent-elicited spectral power and ERP activity associated with noun phrase anaphora, but did not investigate spectral power changes associated with anaphors themselves and is therefore not discussed in this section.

5 Because we did not manipulate the ease with which old or new referents could be integrated (i.e., whether they were semantically coherent with the preceding discourse), our hypotheses primarily focused on the discourse updating processes associated with a new referent.

6 https://osf.io/7pkc5

7 https://osf.io/uak8g/

8 http://meshugga.ugent.be/snaut

9 For data manipulation, analysis and visualization, we used the following packages: dplyr (Wickham et al., 2019), gdata (Warnes et al., 2017), tidyverse (Wickham, 2017), tidyr (Wickham and Henry, 2019), Rmisc (Hope, 2013), ggplot2 (Wickham, 2016), cowplot (Wilke, 2019), lme4 (Bates et al., 2015), lmerTest (Kuznetsova et al., 2017), emmeans (Lenth, 2019).

10 This correction was not pre-registered but requested by a reviewer.

11 The effects in the beta range covered a large number of areas. In order to identify where the effect was strongest, we adopted a conservative cut-off of alpha = 0.005 for data points to be subjected to the permutation analysis. All other settings were identical to those reported in Nieuwland and Martin (2017) and Coopmans and Nieuwland (2019).

12 As pointed out by a reviewer, the observed frontal positive ERP effect may be due to a general unexpectedness of non-repeated nouns (e.g., Van Petten and Luka, 2012), rather than to the introduction of a new referent *per se*. Indeed, a cloze completion test on a subset of 12 items in 12 participants, in which we counted repeated nouns (regardless of a preceding adjective), the expectancy of a repeated noun anaphor was relatively high (72% cloze, range across conditions 69–75%, across items 43–100%, across subjects 50–100%, all cloze data is on our OSF page). In our study, therefore, we cannot distinguish novelty from unexpectedness.

13 We considered the possibility that the reduced N400 for ambiguous nouns is in fact an enhanced positivity associated with easier task performance, but this pattern is difficult to reconcile with the other N400 patterns, such as the smaller N400 for partially matching nouns compared to new nouns despite the fact that partial-match nouns were more difficult to evaluate.
