# Supplementary material for: Distinguishing Old From New Referents During Discourse Comprehension: Evidence From ERPs and Oscillations
Source: Front Hum Neurosci. 2019 Nov 14;13:398. doi: 10.3389/fnhum.2019.00398 (PMC6870011; doi:10.3389/fnhum.2019.00398)

Critical nouns

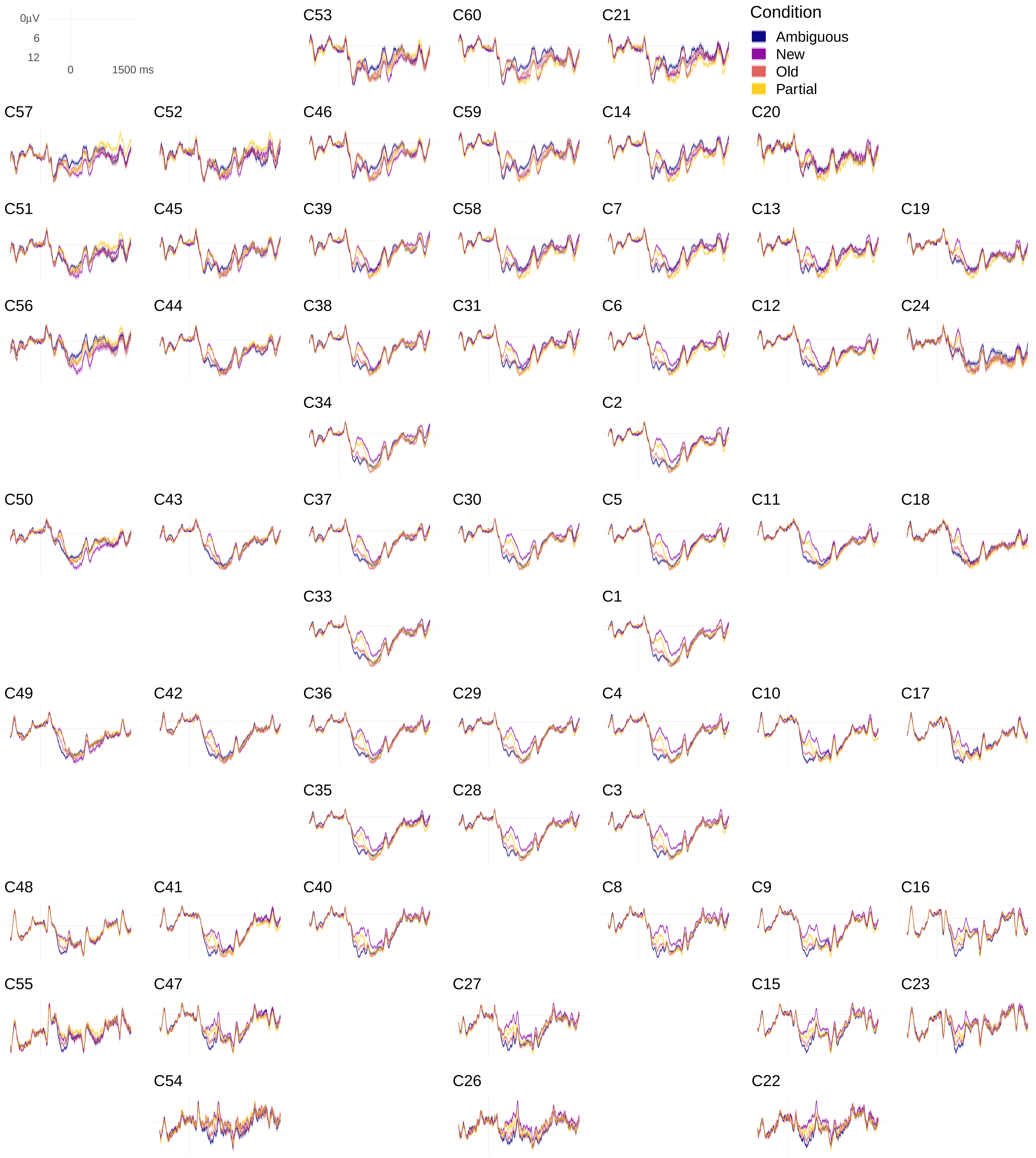

Supplement: Supplementary file 1 [file Image_1.pdf]
