# Supplementary figures and images for: Distinguishing Old From New Referents During Discourse Comprehension: Evidence From ERPs and Oscillations
Source: Front Hum Neurosci. 2019 Nov 14;13:398. doi: 10.3389/fnhum.2019.00398 (PMC6870011; doi:10.3389/fnhum.2019.00398)

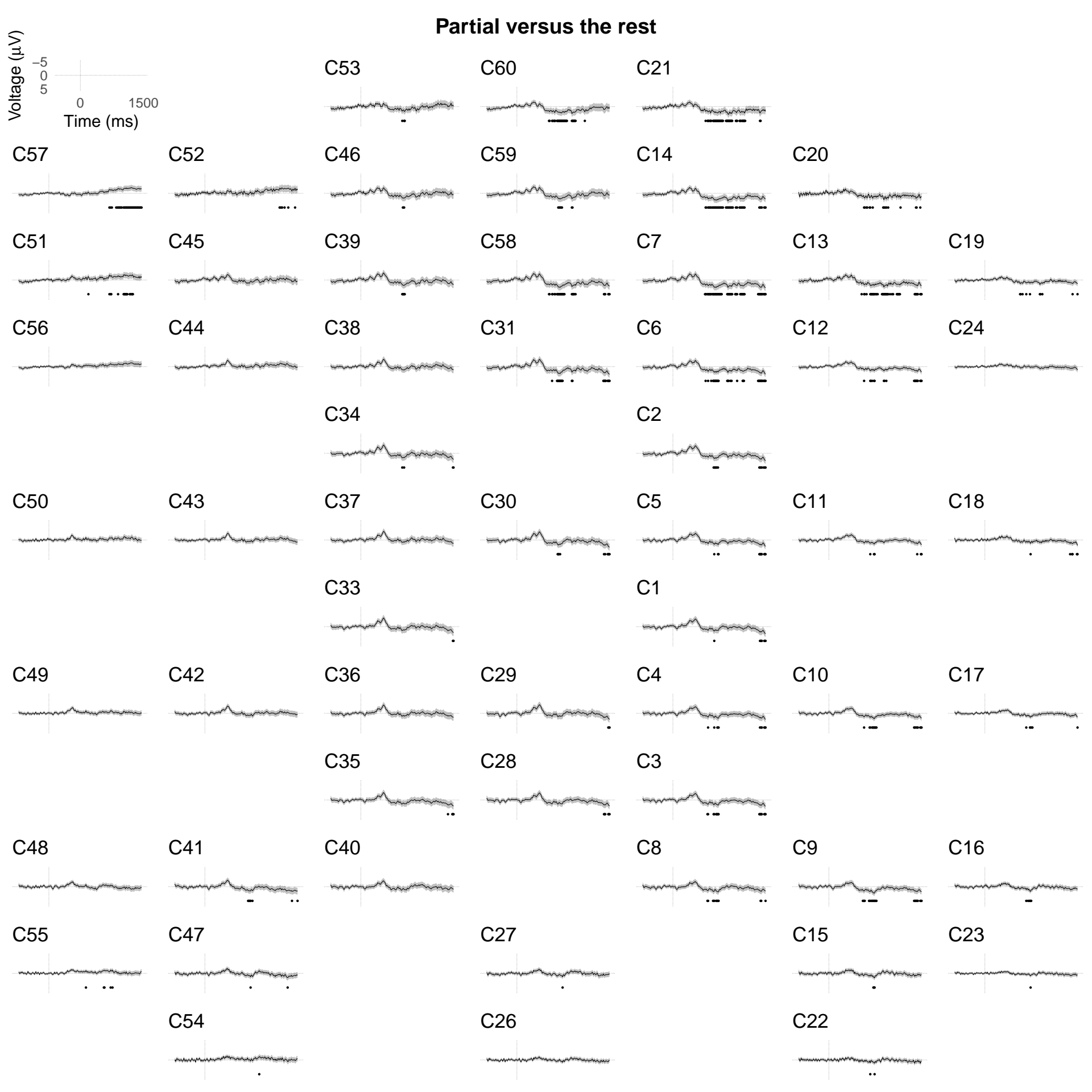

Supplement: Supplementary file 2 [file Image_2.pdf]

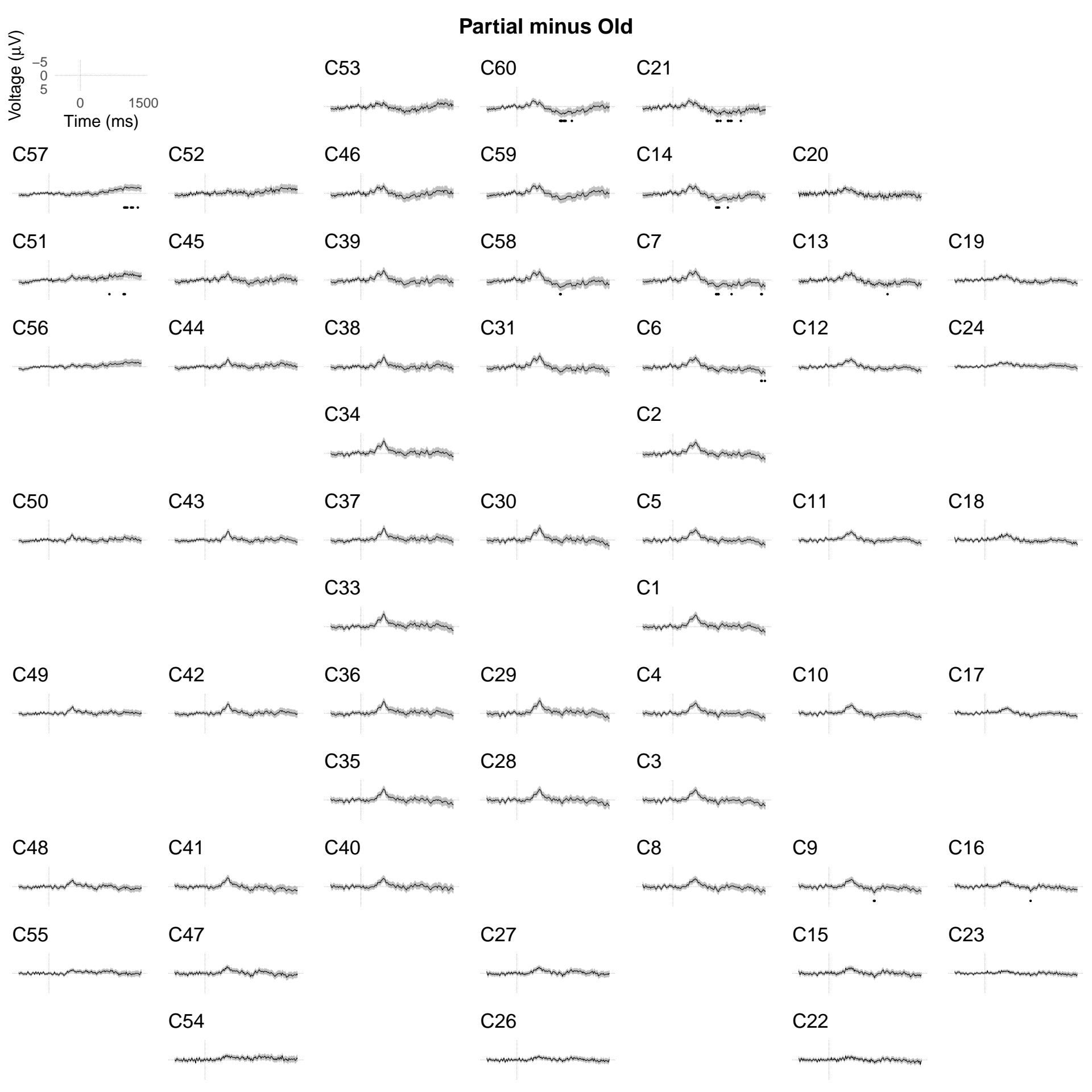

Supplement: Supplementary file 3 [file Image_3.pdf]

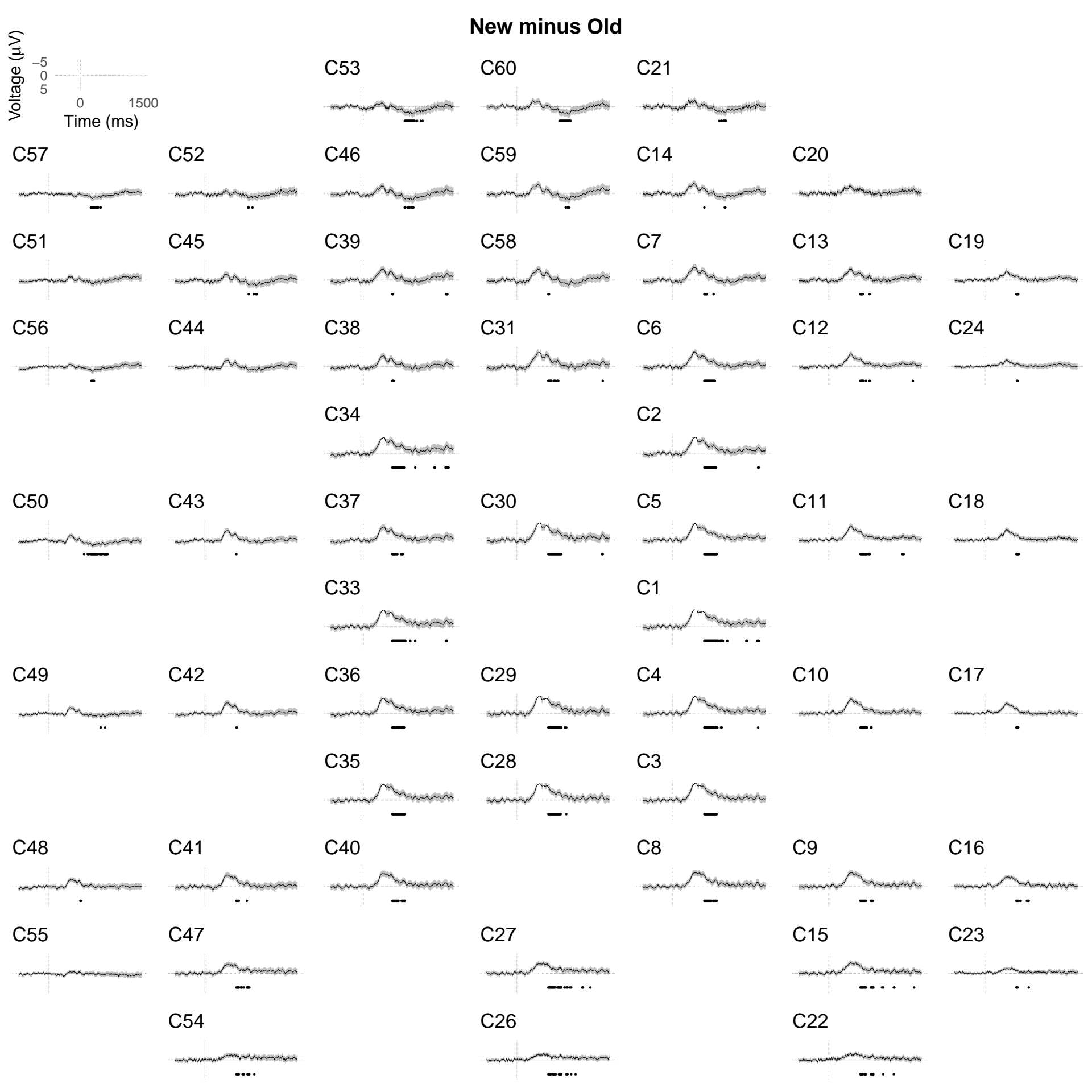

Supplement: Supplementary file 4 [file Image_4.pdf]

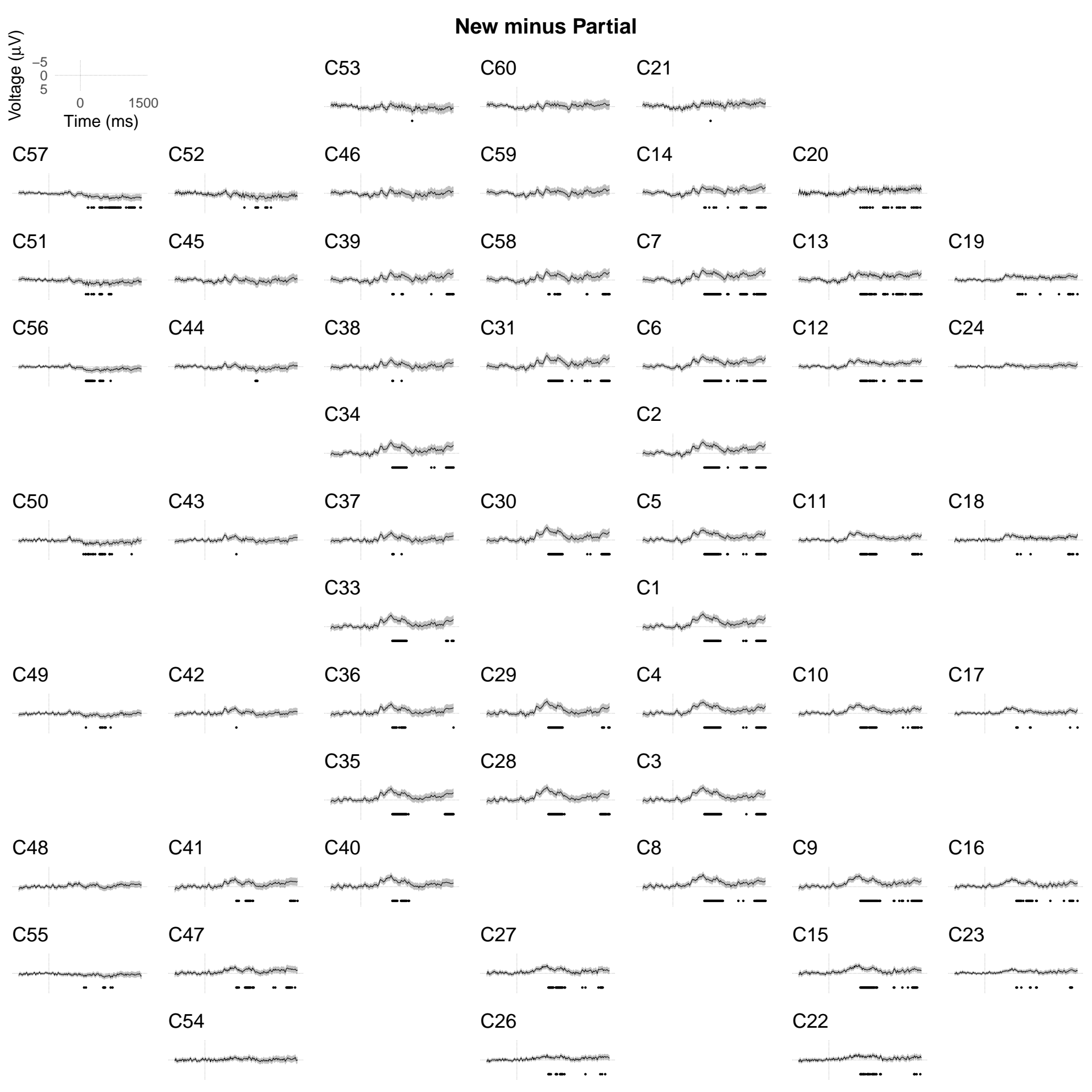

Supplement: Supplementary file 5 [file Image_5.pdf]

# Sentence-final words

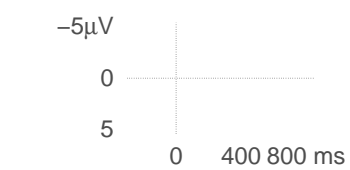

Condition

- Ambiguous
- New
- Old
- Partial

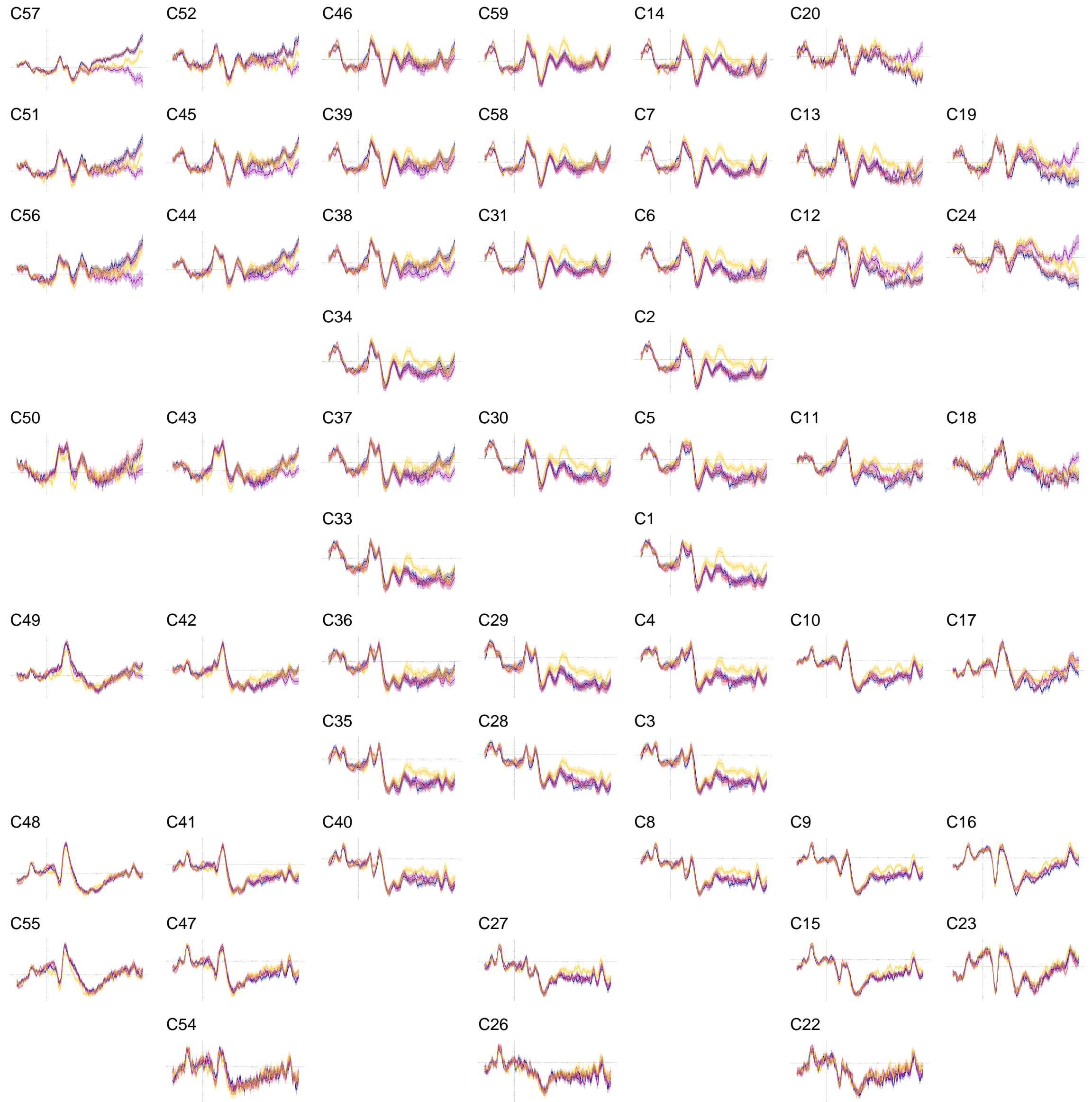

Supplement: Supplementary file 6 [file Image_6.pdf]
