# Supplementary material for: Distinguishing Old From New Referents During Discourse Comprehension: Evidence From ERPs and Oscillations
Source: Front Hum Neurosci. 2019 Nov 14;13:398. doi: 10.3389/fnhum.2019.00398 (PMC6870011; doi:10.3389/fnhum.2019.00398)
Supplement: Supplementary file 7 [file Data_Sheet_1.docx]

| **Supplementary Table 1.** Time-frequency effects in the unconstrained (2-30 Hz) time-frequency analysis of the 200-1500 ms time window after critical noun onset. | | | | |
| --- | --- | --- | --- | --- |
|  | Frequency | Cluster *t*-value | Cluster size | *p*-value |
| Old – New 1  Old – New 2 | 10-15 Hz  0-8 Hz | 24888  -23148 | 12375  3650 | 0.016/0.056  0.016/0.056 |
| Old – Partial | 10-15 Hz | 34393 | 12375 | 0.014/0.056 |
| Old – Ambiguous | 0-15 Hz | -24548 | 8835 | 0.008/0.04 |
| Ambiguous – New | 10-15 Hz | 57649 | 20991 | 0.002/0.014 |
| Ambiguous – Partial | 10-15 Hz | 64256 | 21782 | 0.002/0.014 |
| Partial – New | - | -2673 | 978 | 0.969/0.969 |
